# Biochar as Alternative Material for Heavy Metal Adsorption from Groundwaters: Lab-Scale (Column) Experiment Review

**DOI:** 10.3390/ma17040809

**Published:** 2024-02-07

**Authors:** Paolo Viotti, Simone Marzeddu, Angela Antonucci, María Alejandra Décima, Pietro Lovascio, Fabio Tatti, Maria Rosaria Boni

**Affiliations:** 1 Department of Civil, Building and Environmental Engineering (DICEA), Faculty of Civil and Industrial Engineering, Sapienza University of Rome, Via Eudossiana 18, 00184 Rome, Italy; simone.marzeddu@uniroma1.it (S.M.); angela.antonucci@uniroma1.it (A.A.); mariaalejandra.decima@uniroma1.it (M.A.D.); lovascio.1876149@studenti.uniroma1.it (P.L.); mariarosaria.boni@uniroma1.it (M.R.B.); 2 National Centre of Waste and Circular Economy, Italian Institute for Environmental Protection and Research (ISPRA), Via Vitaliano Brancati 48, 00144 Rome, Italy; fabio.tatti@isprambiente.it

**Keywords:** adsorption, biochar, biomass valorisation, circular economy, groundwater contaminants, heavy metals

## Abstract

The purpose of this manuscript is to present a review of laboratory experiments (including methodology and results) that use biochar, a specific carbon obtained by a pyrolysis process from different feedstocks, as an alternative material for heavy metal adsorption from groundwater. In recent years, many studies have been conducted regarding the application of innovative materials to water decontamination to develop a more sustainable approach to remediation processes. The use of biochar for groundwater remediation has particularly attracted the interest of researchers because it permits the reuse of materials that would be otherwise disposed of, in accordance with circular economy, and reduces the generation of greenhouse gases if compared to the use of virgin materials. A review of the different approaches and results reported in the current literature could be useful because when applying remediation technologies at the field scale, a preliminary phase in which the suitability of the adsorbent is evaluated at the lab scale is often necessary. This paper is therefore organised with a short description of the involved metals and of the biochar production and composition. A comprehensive analysis of the current knowledge related to the use of biochar in groundwater remediation at the laboratory scale to obtain the characteristic parameters of the process that are necessary for the upscaling of the technology at the field scale is also presented. An overview of the results achieved using different experimental conditions, such as the chemical properties and dosage of biochar as well as heavy metal concentrations with their different values of pH, is reported. At the end, numerical studies useful for the interpretation of the experiment results are introduced.

## 1. Introduction

Water is known to be essential for life [1,2]; on the Earth’s surface, it is irregularly distributed, with 97% being saline and 3% being freshwater. Moreover, 69% of freshwater is found in glaciers and ice caps in the Arctic and Antarctic, with another 30% as groundwater, while less than 1% is surface water (lakes, rivers, and swamps), and a small portion (0.001%) is found in vapours, clouds, etc. [3].

Even if surface water is widely used, groundwater is the main source of agricultural and drinking water in many countries where surface water is scarce [4,5]. For example, in some countries with arid climates, such as the Middle East and Saudi Arabia, groundwater is the only water supply. Also, in many European countries (Austria, Belgium, Denmark, Hungary, Romania, and Switzerland), groundwater accounts for more than 70% of the total water consumption. In addition, cities such as Budapest and Rome rely almost entirely on groundwater for their water supply [6]. On the other hand, groundwater is also preferred because the water source is often close to consumers [7,8].

This having been said, it is often difficult to determine the limits of groundwater contaminants. Heavy metals have been widely studied due to their potential toxic effect on living organisms [9,10,11]. Moreover, heavy metals are not degradable by biological or photochemical activities and remain in the environment for hundreds of years [12].

The sources of heavy metal contamination are natural and anthropogenic. In the earth’s crust, heavy metals can be solubilised through natural processes or by a change in soil pH [9,13,14]. Furthermore, groundwater can be contaminated by anthropogenic sources such as landfill leachate [15], sewage, leachate from mine tailings, and seepage from industrial waste [9]. Heavy metals can also be generated by human activities; most of the pollution comes from industry, agriculture, pharmaceuticals, and households [16].

To avoid damage to the environment and public health, heavy metal concentrations in groundwater frequently need to be decreased; thus, various treatment methods have been developed. They are classified as follows: in situ, on-site, and off-site treatments. Physical containment is generally the cheapest technology, but it only avoids dispersion of the contaminant, leaving the contaminants in the site without treatment [17].

In situ technologies (i.e., reactive barriers) can be effective but generally are expensive technologies due to the use of specific materials (i.e., iron) [18]. Moreover, phytoremediation can be effective, but it takes a long time and is imposed by laws.

Ex-situ technologies such as coagulation/precipitation, ion exchange, reverse osmosis, and adsorption processes have been widely investigated by Uwamarriya [7]. They are not easy to operate when dealing with groundwater due to the high volumes involved.

On site technologies can be sometimes attractive for remediation, like the extraction and treatment of water with its subsequent eventual reintroduction in the soil. In this case, several technologies are the same as the ex-situ ones, and between them, adsorption is one of the most used and effective to reach the requested values of remediation.

Activated carbon (AC) is one of the most used adsorbents for the removal of contaminants in water due to its properties. AC is primarily prepared from coal, coconut shells, lignite, and wood, and activated by physical and chemical methods. Due to its high specific surface area, chemical stability, durability, high capacity of adsorption, and not selective adsorption capacity, AC has been widely used to remove heavy metals from groundwater [19,20,21]. However, the regeneration costs of AC may limit its extensive use [18,22]; therefore, it is important to develop low-cost adsorbents with a high adsorption capacity for the removal of pollutants from aqueous systems [23].

Adsorption onto biochar (BC) is generally considered one of the most cost-efficient and effective treatment methods for removing heavy metals in water and soils [24], and it could represent an alternative low-cost and sustainable adsorbent for contaminant removal from water [18,25]. Biochar is a carbon-rich solid material produced by the thermal decomposition of organic material with a limited supply of oxygen (pyrolysis). It can be produced sustainably under controlled conditions and with clean technologies [26].

BC is produced from various types of wastes such as woody biomass, animal manure, waste paper, and sludges [25,27,28]; it is sometimes also considered a solid by-product, which causes problems in its final disposal. The specific properties of biochar, including its large specific surface area, porous structure, enriched surface functional groups, and mineral constituents, allow it to have a high adsorption capacity [29]. Moreover, BC is easier to prepare and less expensive than active AC or other adsorbing materials [30]. Biochar has a similar porous structure to activated carbon, which is the most widely used and efficient sorbent in the world for removing various pollutants from water. Compared to activated carbon, biochar appears to be a new potential low-cost and effective adsorbent because the cost of biochar is six times lower than activated carbon, due to its lower energy requirements and the fact that it can be used without chemicals or physical activations [31].

Due to specific conditions (i.e., the presence of other competitor substances, the heterogeneities of the media, and biochar’s specific characteristics), an experimental preliminary phase is always required to evaluate the parameters and to verify the suitability of the use of the biochar in that specific context.

The aim of this manuscript is to provide a review of laboratory-scale column systems used to identify the physic-chemical biochar properties and operational parameters of column tests that could be useful for an effective adsorption process.

Furthermore, the research results are presented for a fast consultation if needed. the approach presented in this paper is obviously finalised to contribute to the broadening and extension of the knowledge on the procedures adopted for the lab tests in the context of environmental sustainability when dealing with the use of an absorbent material like biochar.

## 2. Biochar

Biochar, which has been known since olden times for its beneficial effects on soil, is produced using the thermal treatment of organic residues from different sources conducted under controlled conditions, i.e., without an oxidising agent [32,33,34,35,36]. Moreover, biochar is a type of specific charcoal that can be obtained by the pyrolysis processing of biomasses with a limited supply of oxygen [37,38,39] and with clean technology [26,40]. In fact, the International Biochar Initiative (IBI) defines biochar as a solid material produced by the thermochemical conversion of biomass in an oxygen-limited condition [41]. Moreover, Hagemann et al. [42] underlined the difference between charcoal and biochar: biochar is produced for agricultural use with the intent to be applied to soils as a means of improving soils productivity and physical properties [38], such as water and nutrient retention, and of carbon storage [32], while charcoal is mostly used as a fuel for cooking and heating [43] and is commonly associated with barbequing [44].

The specific properties of biochar, including its large specific surface area (S_BET_), porous structure, enriched surface functional groups, and mineral constituents, enable it to have a high adsorption capacity [29]. The density and size of its pores, which are generated by the volatilisation of organic substances, depends on the feedstock and on the temperature during pyrolysis [45]. The main biochar characteristics can be also determined by specific analytical techniques as indicated in Table 1.

The production of biochar is a process that allows for the valorisation of materials that are substantially considered waste. The Food and Agriculture Organization of the United Nations (FAO) reports that one billion tons of food are wasted every year, of which 60% is solid food waste, such as fruit and vegetable scraps, including peels, seeds, and pips, posing a serious disposal problem [29]. To promote a zero-waste strategy, it is important to highlight the importance of biochar in the circular economy [47]. The transformation of waste into value-added products is one of the alternative solutions to minimise the problem of waste production [48]. In fact, the use of biochar as an environmental application can lead to a reduction in agricultural waste [49] and plant biomass used in the pyrolysis process [50].

**Figure 1:**
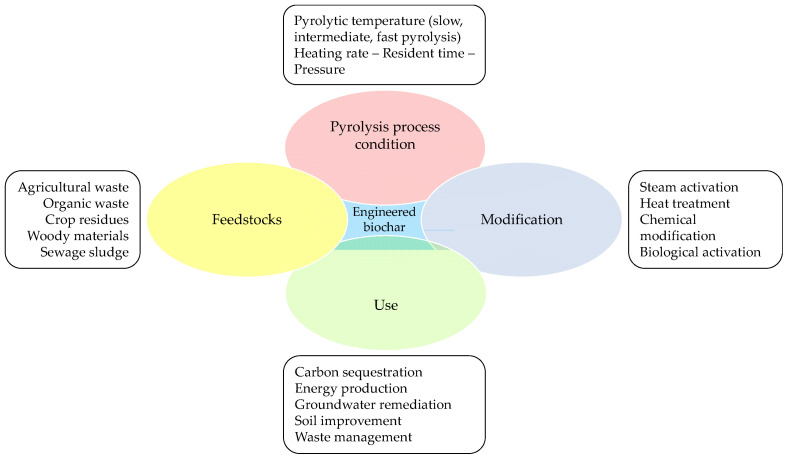
Biochar: origin, preparation, modification, and use.

Biochar is not only an effective material for environmental remediation but can also be used in other fields [51,52]. In environmental management, biochar can be used for several purposes, as shown in Figure 1, including the following: the improvement of soil quality [53,54], greenhouse emission reduction (mainly CO_2_), climate change mitigation [55,56], waste and heavy metals management [57,58,59,60,61,62], and as adsorbent material for the removal of heavy metals from contaminated water [63,64,65,66,67].

The biochar applications shown in Figure 1 are in addition to its well-known use for modifying and improving the chemical–physical characteristics of soils, as reported in [68,69,70,71,72].

In fact, biochar bulk density is much lower than that of soil [68]; therefore, the introduction of biochar into soil reduces its bulk density proportionately to the amount of biochar used, with soil bulk density being related to the fertility and hardness of the soil.

Tan et al. [69] reported also that the cation exchange capacity (CEC) of the soil is increased by biochar due to the action of oxygen-active groups lying on the surface of biochar that absorbs H^+^ and modifies soil pH. Moreover, the soil organic matter (SOM), which is the source of nutrients of the soil, increases with the addition of biochar [69].

O’Neill et al. [70] explained that biochar also promotes the transformation of N into the soil and increases the organic matter (OC) content of the soil. The total P increase if the pyrolysis temperature is greater than 300 °C.

Furthermore, biochar increases the soil porosity, thus positively changing soil conditions and promoting plant growth, as reported by Oguntunde et al. [71].

Regarding the pH, Novak et al. [72] explained that the P, Ca, Mg cations of biochar can react with the exchangeable soil ions (Al and H^+^), causing a decrease in soil acidity.

Therefore biochar has several distinguishing points, and among them, they also have an influence on greenhouse gases in soil [73,74].

The main properties/applications that characterise this specific material in the above-mentioned process can be briefly summarised as follows [75]:

- Formation of aromatic stable carbon structure of biochar and silica–carbon complexes;
- Formation of complexes between biochar and soil minerals that reduce the microbial decomposition of biochar;
- Absorption of soil organic matter with the formation of aggregates that contrast degradation;
- Biochar modification of soil enzyme activities;
- Change in soil pH which changes the amount of NO_2_ that turns into N_2_;
- Increase in microbial communities that carry out denitrification;
- Increased absorption of NH_4_^+^ and NO_3_^−^ ions by soil;
- Soil aeration which decreases denitrification.

The studies available on biochar should be appropriately used to select the most suitable one to be used in environmental projects; furthermore, the possibility of using an engineered procedure able to select increasingly specialised biochar for various topics must be taken into consideration [35,76]. For this purpose, it is important to know in detail the operating conditions and the functionality of biochar. This means obtaining accurate knowledge on the material, beginning with the primary resources used in its production, continuing to the technologies used in its manufacture, and ending with the potential alterations it can undergo. For this reason, the application at the field scale of this material for adsorbent purposes needs to undergo a preliminary phase at the lab scale, from which the characterising parameters can be derived for its use in the optimal design of an intervention.

### 2.1. Feedstock and Production Process

The feedstock and pyrolysis temperature are the main responsible for biochar’s physical-chemical properties [77,78]; in fact, pyrolysis is the most common treatment to produce biochar.

Pyrolysis is a thermochemical decomposition process achieved when heat is applied in the complete absence of an oxidising agent (usually oxygen), with the subsequent breaking of the original chemical bonds [79]. Depending on the residence time, pyrolysis can be categorised as fast, intermediate, or slow, influencing biochar, bio-oil, and syngas production quantities [80,81].

Table 2 shows the different process-operating conditions and an average value of the different product yields.

As highlighted in Table 2, slow pyrolysis is carried out with a large range of residence time and a heating rate ranging between 0.1 and 0.2 °C/s, leading to different product yields. Pariyar et al. [83] suggested that biochar, when produced by pyrolysis, is suitable for carbon sequestration and agricultural purposes. Pyrolysis carried out at a high temperature ranging from 500 to 1000 °C and at a faster heating rate of 10–200 °C/s causes the fast decomposition of biomass and the generation of vapours and aerosols with some biochar. Fast pyrolysis occurs with a short residence time (i.e., t = 0.5–2.0 s) and with fine-particle-size (i.e., D < 1 mm) feedstock [84]. Moreover, fast pyrolysis favours the production of bio-oil (i.e., η = 75%) [85]. On the other hand, slow pyrolysis employs a residence time ranging from hours to days, and it is produced at a moderate temperature (i.e., T = 100–550 °C) resulting in higher biochar yield (i.e., η = 35%) compared with fast pyrolysis (i.e., η = 12%) or intermediate pyrolysis (i.e., η = 25%). 

Several types of organic biomass can be used to obtain biochar. The feedstock is classified into three types of biomasses: plant, manure, and residual source [86].

Hemicellulose, cellulose, and lignin are the main components of the feedstock’s organic matter [87]; these materials strongly influence the characteristics of the biochar mainly due to their degradation that occurs at different temperature ranges (i.e., hemicellulose, cellulose, and lignin, respectively, degrade at 200–260, 240–360, and 280–350 °C) [88].

The raw materials are obtained from agricultural biomass, which is one of the most abundant renewable resources on earth and therefore the most economical and readily available, such as rice husks, orange or banana peels, peanut shells, sugarcane bagasse, and dried pulses [89,90]. Other feedstocks have also been used to produce biochar through various thermochemical processes, including crop residues, woody biomass, animal bedding, and solid waste [91].

Many of the biochars that are used for water contaminant removal are prepared from local agricultural waste [92].

Table 3 shows the main feedstock and production processes depending on the raw material. Specific operating conditions should be chosen to obtain high-quality biochar.

As highlighted in Table 3, Xue et al. [94] and Hu et al. [95] produced hydrobiochar and biochar from peanut hulls.

Tabassum [93] used the BC derived from an orange peel, banana peel, and rice husk mixture to treat As-contaminated groundwater. Biochars studied by Boni et al. [99] and Beiyuan et al. [100] are other examples of agricultural residue transformed in biochar for remediation purposes.

Another possible source is the sludge derived from civil wastewater treatment plants; the disposal of this residual is a particular concern, and it is linked to serious environmental issues. As a result, the pyrolysis of sewage sludge into biochar could be a promising alternative disposal method; according to Zhou et al. [107], sewage sludge biochar application reduces heavy metal mobility in co-contaminated soil.

A large number of similar example data are reported in most of the scientific literature on biochar [46,56,82,108,109,110]. Ahmad et al. [111] provide a statistical summary of the characteristics and parameters of biochar at different pyrolysis temperatures (Table 4).

Moreover, the modifications caused by the pyrolysis process on the raw material are important [112]. By selecting the feedstock and the operating conditions, it is possible to obtain engineered biochar that can be used for specific purposes, including remediation [113]. Table 5 summarises the main characteristics of biochar.

The data in the scientific literature indicate that the trends of some characteristic parameters of biochar are dependent on the pyrolysis temperature. For example, a higher pyrolysis temperature corresponds to a higher amount of C and a lower percentage of H and O; therefore, the molar ratios of H/C and O/C decrease and thus decrease the hydration and oxygenation of biochar, increasing its aromaticity [109,111]. Furthermore, according to Wang et al. [130], with an increase in temperature, the surface area, the volume of the micropores, and the pH of biochar increase. 

The relative proportion of biochar components determines the chemical and physical behaviour and function of biochar, as well as its transport and fate in the environment. The ash content (A) generally ranges from 0.5 to 5.0%, and this value depends on the biomass feedstock. A higher ash content is present if the feedstock is sewage sludge [131], grass, or grain husks or manure, while it is lower if the raw material is wood [132]. Moisture is an important component of biomass feedstock because higher moisture contents increase biochar production and transportation costs [132]. According to Verheijen et al. [74], keeping the moisture content up to 10% (by weight) is preferable to dry raw biomass.

The elemental composition of the biochar includes several elements like C, H, O, N, S, P, K, Ca, Mg, Na, and Si [111]; however, during the pyrolysis, the percentage of chemical elements sometimes undergoes a significant change [87].

Table 6 reports the chemical composition and the main properties of crops before and after slow pyrolysis, specifically concerning corncobs, cassava rhizomes, and cassava stems.

Table 6 shows an increase in C (wt%) after the transformation of the feedstock into biochar. In the three types of biochar analysed, H (wt%) and O (wt%) decrease, while the content of N (wt%) increases. The molar ratios of H/C and O/C decrease, while the molar ratio of C/N shows a different trend for the three crops. There is an evident increase in the specific surface area (S_BET_) and in the total pore volume (V_T_), while there is a slight decrease in the average pore diameter (d).

The pyrolysis temperature also affects the pH and the value of the cation exchange capacity (CEC) of biochar; the pH increases with pyrolysis temperature because it is related to ash content [108]. On the other hand, the CEC value decreases as the pyrolysis temperature increases because of the loss of functional groups from the surface of biochar [83,133]. Functional groups such as carboxylic, amino, and hydroxyl are some of the parameters that determine the absorption capacity of metals by biochar [109].

### 2.2. Biochar Activation and Modification

The effectiveness of biochar in the various environmental fields can be increased by modifying its characteristics through chemical modification or physical/thermic activation. Some of the common modification methodologies are shown in Table 7.

The activation methodology must be chosen based on the required effects. In the remediation of unsaturated soils, the type of contaminant, the environmental situation, and the final goal that has to be reached must be considered. It may be necessary, in specific cases, to modify biochar, for example, using a technology that allows for the increase in the functional groups on its surface and pore volume to enhance the adsorption capacity by means of, for example, magnetic or steam modification [31].

In aqueous solutions, instead, the inner and outer share complexation, the electrostatic interaction, the ionic exchange, and precipitation must be considered, so physical or chemical activation are the most suitable methods that can be used to develop these removal mechanisms [130]. Table 8 shows different ways to produce modified biochar. Engineered biochar is specifically designed to perform specific functions.

## 3. Heavy Metals

Heavy metals are defined as a group of metals and metalloids with a higher density than water [141,142] and a toxic or poisonous effect on humans or the environment at low concentrations [143]. Heavy metals include metals with a density at least five times greater than that of water (i.e., about 5.0 g/cm^3^), and some metalloids, such as arsenic [144]. Based on a literature review, chromium, lead, cadmium, iron, arsenic, cobalt, mercury, copper, and zinc are considered heavy metals [145].

The presence of heavy metal contamination in groundwater is well known due also to natural phenomena such as the erosion and weathering of parent rocks [146]. Natural events such as volcanic eruptions, soil erosion, the rock cycle, atmospheric influences, and tides contribute to the natural cycle of metals, so they reach several environmental compartments, including water, soil, and air [147]. At the same time, groundwater is often contaminated with heavy metals from anthropogenic sources like landfill leachate, sewage, excavation activities, and the uncontrolled disposal of industrial waste [15,16].

The toxicity, mobility, and reactivity of heavy metals depend on their oxidative states, which are influenced by pH, Eh, and temperature [9,15]. Several previous studies reported that the interaction of heavy metals with microorganisms reduced the expression of several enzymes [148,149,150,151]. Furthermore, some heavy metals, at high concentrations, become toxic because they interact with metal-sensitive enzymes, causing the death of some organisms [142]. Another consequence is bioaccumulation in organisms because heavy metals are not easily metabolised [10,14,148,152,153]. Some of the heavy metals easily encountered in remediation processes are quickly described in Table 9, and for each one, the concentration limit at which they are considered dangerous is reported [1]. This review will focus on the potential interactions between the identified heavy metals and biochar.

## 4. Applications at Lab Scale of Adsorption Process for Heavy Metal Removal

### 4.1. Adsorption Process

The treatment of groundwater contaminated by heavy metals is considered an international challenge [202,203,204,205,206,207]. To restore groundwater contaminated by heavy metals, several remediation technologies have been developed, such as chemical precipitations [208], ion exchange [209], electrokinetic technology, redox methods [210], membrane technologies [211], and permeable reactive barriers [212]; however, the use of these technologies has several contraindications [20,213,214,215]. Therefore, interest in environmentally friendly and economically acceptable treatment technologies for sustainable groundwater remediation [216,217] is growing.

Adsorption is a widely applied technique for removing heavy metals from groundwater [218]. Today, several new adsorbents, such as activated carbon [219], nanotubes [220,221], multi-material nanoparticles, and biochar are being studied as potential sorbents [222,223,224,225]. Adsorption is a chemical–physical phenomenon consisting of the accumulation of one or more fluid substances (liquid or gaseous) on the surface of a solid condensate. In the phenomenon of adsorption, a chemical–physical interaction occurs between chemical species (molecules, atoms, or ions) on the interface between two distinct phases.

**Figure 2:**
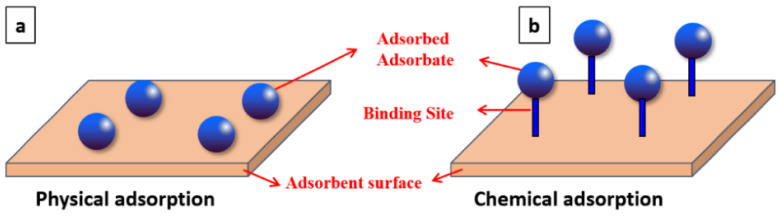
Physical adsorption (**a**) and chemical adsorption (**b**) by Ho [229].

The species subjected to adsorption is called the adsorbate, and the solid phase is called the adsorbent [226]. From a thermodynamic point of view, it can be stated that adsorption is a spontaneous process (ΔG < 0) and is characterised by a decrease in the entropy of the adsorbed substance incorporated into the solid (ΔS < 0). Adsorption is an exothermic phenomenon (ΔH < 0) and is therefore favoured by low-temperature values; the amount of heat generated by the process is a function of the type of bonds formed [227]. Depending on the nature of the interactions that occur between the adsorbate and the adsorbent, and thus on the extent of the energy of the bonds with which the particles are retained on the surface, adsorption can be defined as physical, also called physisorption, or chemisorption [228]. Figure 2 shows a schematisation of physical and chemical adsorption.

Physical adsorption is characterised by weak intermolecular bonds, such as electrostatic or van der Waals, due to the polarity of the adsorbed molecules and the presence of positive or negative ions on the adsorbent surface [230]. Chemical adsorption is, on the other hand, characterised by strong intramolecular bonds, a specific phenomenon that occurs at active sites capable of forming bonds with the molecules of the liquid [231].

The specific behaviour of both processes and the eventual modification in electron density are presented in [145,228,232,233,234].

Adsorption is a superficial process [235]. For this reason, adsorbent materials must have a high specific surface area, which refers not only to the size of the granules of which they are composed but also, and more importantly, to the internal porosity (p) of those granules [236]. The series of treatments by which adsorbents are prepared results in the formation of pores of different sizes [237]. In the case of biochar after its pyrolysis process, this material can be activated, which means that its specific surface area is increased by contact with a stream of air, CO_2_, and water vapour to increase adsorption.

The factors that influence the kinetics of the adsorption process are mixing intensity, temperature, pH, and the properties of the adsorbent and the adsorbate [238]. 

When adsorbents are saturated and no longer able to remove the pollutant [29], the spent adsorbents can be regenerated to restore their adsorption properties or replaced with new ones, while disposing of the spent one. The regeneration process is carried out by heat treatments in which the adsorbate is oxidised and thus removed from the adsorbent material [96].

### 4.2. Column Systems

**Figure 3:**
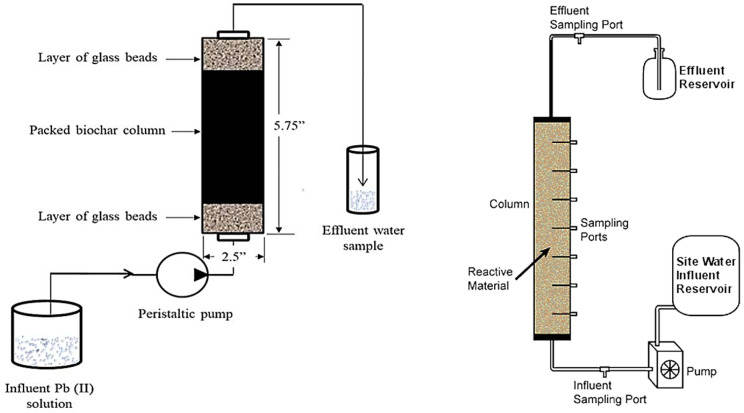
Schemes of possible fixed-bed adsorption systems [240,241,242].

Studies on adsorption processes at the lab scale can be carried out with two different types of reactors: batch (i.e., discontinuous mode) or column (i.e., continuous flow) [239]. Column systems with continuous flow, as shown in Figure 3, are generally used for this kind of experiment. The flow can be upflow or downflow and typically governed by pump systems.

Flow conditions within the column are described by two hypotheses: complete mixing in the transverse direction and the lack of mixing in the longitudinal direction (laminar flow conditions) [243,244]. In both cases, the hydraulic residence time of the liquid in the device is chosen to achieve conditions sufficiently close to thermodynamic equilibrium [238], and steady-state conditions are also generally assumed.

**Figure 4:**
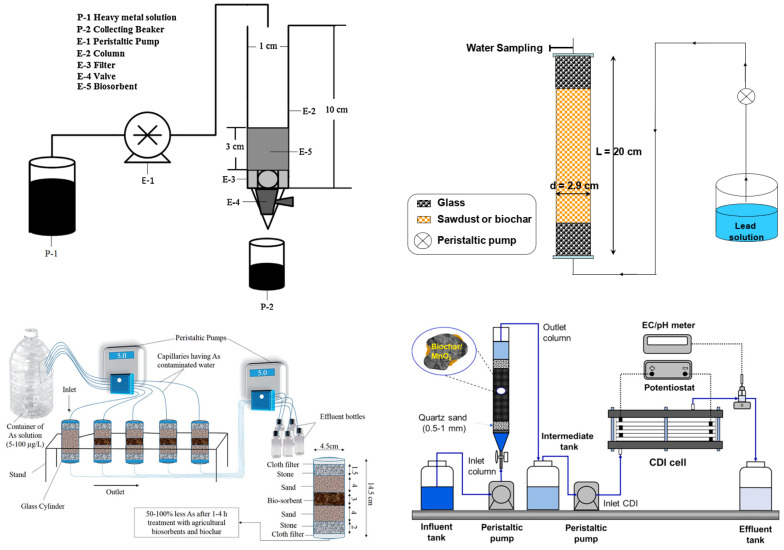
Examples of experimental setups in different studies [93,249,250,251].

Therefore, in continuous flow processes the adsorbent solid may be arranged as a fixed bed or as a moving bed in contact with the liquid, while the liquid flow may be descending or ascending [245,246,247,248]. Figure 4 below presents different configurations of the experimental apparatuses used for heavy metal removal through fixed-bed columns and biochar.

Adsorption modelling processes are carried out through the definition of adsorption isotherms, i.e., empirical relationships that relate the number of species adsorbed on the solid per unit mass of the solid to the concentration of the same species in solution at a given temperature and under conditions of thermodynamic equilibrium [252,253].

#### Breakthrough Curves

**Figure 5:**
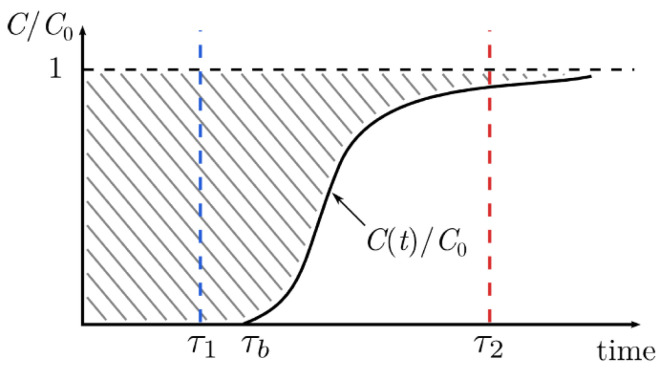
Schematic of a breakthrough curve [255].

Studies using column systems can be finalised using models based on the interpretation of breakthrough curves [254]. Breakthrough curves, as shown in Figure 5, describe the dynamics of the adsorption process and provide relevant information and key parameters for the design, operation, and optimisation of the separation system.

The breakthrough curve is characterised by two characteristic times: breakpoint (t_b_) and exhaustion time (t_e_). The breaking point identifies the time in which the solute begins to leave the column, i.e., the concentration in the fluid phase begins to increase. The saturation time characterises the time after which the adsorption process stops due to all the sites on the adsorbent becoming saturated. This is coincident with the time of the exhaustion point, and the concentration of the outgoing solution has the same concentration as the one supplied [238]. Further conditions about the column behaviour can be found in [256].

## 5. Biochar: Experiments and Results at Lab Scale

### 5.1. Main Removal Mechanisms of Heavy Metals Using BC

The following section is an overview of the main findings reported in the scientific literature on the use of biochar as an adsorbent in column systems. Table 10 summarises the biochar mechanisms for heavy metal removal concerning specific biochar properties.

Some biochar types and the remediation mechanism of specific heavy metals is shown in Table 11.

The column tests, according to the different heavy metals studied and the specific biochar used, have shown different rates of removal and adsorption capacity. Studies have been carried out considering different aspects, such as the feedstock properties of raw materials used for biochar production. The results have allowed for the definition of the corresponding control parameters by means of the curve breakthroughs simulated using different models.

### 5.2. Feedstock Properties: Influence on the Removal Efficiency

Table 12 shows removal efficiency, R (%), and absorption capacity, q (mg/g), for different kinds of feedstock depending on the initial concentration and bed volume.

The data in Table 12 show, as expected, how the adsorption capacity can present a broad distribution depending on the starting material used and on the starting concentration of the metal.

Several examples are present in the literature about these aspects. For example, Pb removal efficiency by AMBIOTON^®^ and RE-CHAR^®^ biochar reported high values [99,101], showing an absorption capacity of 110.73 and 163.89 mg/g, respectively, when the initial concentration was 100 mg/L, while hemp-derived biochar (HB) has an absorption capacity of only 3.32 mg/g at the same initial concentration [104].

BC@MnO_2_-X biochar (biochar loaded with manganese dioxide) has an even greater absorption capacity at the breakthrough point (i.e., q = 2.1 × 10^6^, 1.1 × 10^6^, 6.7 × 10^5^, and 5.2 × 10^5^ mg/g, respectively, for Pb, Cd, Cu, and Zn) than commercial MnO_2_ composites [103]. 

Xue et al. [94] conducted an experiment and developed a model to examine the effect of H_2_O_2_ treatment on hydrothermally produced biochar (hydrochar) from peanut hulls. The measurements showed that the modified biochar had an enhanced Pb sorption ability, so it can be considered comparable to that of commercial activated carbon.

Tabassum et al. [93] showed that the As concentration in the groundwater water after treatment was below the WHO safe limit for drinking water (10 mg/L), indicating that these biosorbents and biochar in particular can be considered effective for the clean-up of As-contaminated drinking water. Wei et al. [96] showed that although Fe-based biochar adsorbents are attractive for removing As from water due to their low costs, practical applications of these granular adsorbents are limited by slow adsorption kinetics.

Liang et al. [98] proved the hypothesis of simultaneous remediation of both heavy-metal-contaminated soil and groundwater by integrating the chemical immobilisation as well as the pump and treat methods. 

Boni et al. [102] compared beech charcoal produced by pyrolysis with the same material subjected to bio-activation. Both resulted in potential adsorbents for the remediation of cadmium-contaminated groundwater. The experiment included the determination of the main physical–chemical characteristics of two materials and batch tests for the determination of the kinetics and isotherms of adsorption. Column tests were also conducted, and the results showed that both charcoal and bio-activated charcoal (biocharcoal) are effective adsorbents for Cd.

Beesley and Marmiroli [59] monitored As, Cd, and Zn in contaminated soil amended with biochar and green waste compost over 60 days of field exposure, after which its phytotoxicity was assessed by a simple bio-indicator test. Cu and As concentrations increased more than 30 fold after adding both amendments, which was associated with the significant increases in dissolved organic carbon and pH, whereas Zn and Cd significantly decreased. Among the two amendments, biochar was the most effective, resulting in a 10-fold decrease in Cd and a resultant reduction in phytotoxicity.

Zhang et al. [103] used a redox precipitation method to load manganese dioxide (MnO_2_) nanoparticles onto biochar (BC) (BC@MnO_2_) pyrolysed from invasive water hyacinth, and the adsorption of Cd(II), Cu(II), Zn(II), and Pb(II) was investigated. The results revealed that the BC surface was covered by many intertwined thin amorphous MnO_2_ nanosheets, which significantly increased its specific surface area and pore volume. The adsorption of heavy metal ions by BC was negligible, whereas the MnO_2_-containing adsorbents exhibited a high capacity for adsorbing heavy metal ions.

Ding et al. [104] obtained engineered biochar through the slow pyrolysis of hickory wood that was further modified with NaOH. After modification, the biochar’s surface area, cation-exchange capacity, and thermal stability were significantly improved. The modified biochar exhibited a much larger (2.6–5.8 times) metal adsorption capacity than the pristine biochar, and a test comparing the competitive batch adsorption of mixed metal ions [Pb(II), Cd(II), Cu(II), Zn(II), and Ni(II)] showed the preferential adsorption of Pb(II) and Cu(II) onto modified biochar.

Beiyuan et al. [100] explored the potential use of low-cost adsorbents as PRB media and assessed their longevity and risk mitigation against the leaching of acidic rainfall through an e-waste recycling site, of which Cu, Zn, and Pb were the major contaminants. The authors reported the values of the adsorption coefficient for different types of material and for different isotherms. The results showed that BC was suitable but showed a limited time of action.

Trakal et al. [105] used biochar produced from brewers draff via pyrolysis. The biochar was also activated using a KOH solution (2M) to enhance its sorption efficiency to remove Cu from two different aqueous solutions. Batch sorption and column experiments were used to evaluate the efficiency of both biochar (BC) and activated biochar (BCact) to remove Cu from the solutions. The results showed an increase in the absorption capacity of the activated biochar (i.e., q = 10.33 mg/g) with respect to the non-activated one (i.e., q = 8.77 mg/g).

Park et al. [106] evaluated the adsorption of heavy metals in single- and ternary-metal forms onto chicken bone biochar (CBB). The results from both the batch and column experiments show that competitive adsorption among metals increases the mobility of these metals. The maximum metal adsorption capacity of the metals in the column experiments was higher than that in the batch experiment, indicating that other metal-retention mechanisms could be involved.

Zhou et al. [107] evaluated the effect of sewage sludge biochar on the adsorption and mobility of Cr, Mn, Cu, and Zn. Biochar (BC400) was produced via the pyrolysis of municipal sewage sludge at 400 °C. The adsorption capacities for Mn, Cu, and Zn decreased in the multimetal solution due to competitive adsorption, whereas the capacity for Cr increased. The results showed that surface precipitation is an important mechanism in the sorption of these metals on BC400.

Hu et al. [95] have developed a novel permeable reactive barrier (PRB) filler that was prepared with slow-release nutrient-immobilised biochar (SRN-IB) to remove Cr(VI) from an aqueous solution using *Morganella morganii* subsp. Kinetic studies revealed that the removal effect of *M. morganii* subsp. on Cr(VI) was better than that of ZVI (Zero-Valent Iron), which showed a higher removal efficiency and faster reaction rate.

Furthermore, with respect to the possible leaching of weighed metals from biochar, Zhang et al. [261] explained that the pollutants, both organic and inorganic, already present in the feedstocks remain immobilised within the solid matrix thanks to pyrolysis. In fact, through this thermal convection process, the aromatic structure formed allows for heavy metals to be strongly bonded to the biochar [262].

### 5.3. Physical Properties: Influence on the Removal Efficiency

Variations in physical properties, such as specific surface area, pore size, and volume can have an effect on biochar adsorption [31]. Table 13 shows the absorption capacity, q_m_ (mg/g), for a different kinds of feedstock, depending on the type of activation initial concentration and on bed volume.

Jellali et al. [250] studied the removal of lead by using biochar obtained from raw cypress sawdust (RCS) and then pre-treated with magnesium (Mg-B), which was the result of five treatment steps. The physicochemical characterisation of the adsorbents used shows that the chemical–thermal treatment of RCS significantly increased its specific surface area from 10.5 to 35.0 m^2^/g. The experimental amounts of lead adsorbed by the RCS, about 7.4, 7.8 and 7.9 mg/g, are lower than when treated with Mg-B, which are equal to 77.1, 89.9 and 97.2 mg/g for different initial lead concentrations (25, 50 and 100.0 mg/L).

Another example can be found in Moreno-Pérez et al. [263], in which commercial biochar obtained from the pyrolysis of bovine bones (BBs) was used to remove Cd, Ni, Zn, and Cu. The used biochar had a surface area of 113.3 m^2^/g and a micropore volume of 0.001 cm^3^/g. Simple, ternary, and quaternary solutions of the tested heavy metal ions were used for HM adsorption, and q_m_ decreased in the following order concerning the column feed type: quaternary < ternary < single. The highest adsorption capacities were obtained for copper in single and multimetallic solutions and ranged from 4.23 × 10^2^ to 1.06 × 10^3^ mg/g.

Wei et al. [96] conducted a study on the removal of As(III) and As(V). biochar was prepared from cotton fibres (CFs) obtained from waste. Then, they were pyrolysed in a tubular furnace at 800 °C for 2 h in an atmosphere with nitrogen. Finally, the furnace was cooled naturally to reach room temperature (RT) to obtain biochar, and then it was coated with iron oxide nanoneedles by a hydrothermal reaction (Fe-NN/BFs). It was observed that the specific surface area increased from 2.45 m^2^/g for BF to 8.68 m^2^/g for Fe-NN/BFs. Bare BF showed no adsorption of As(V) and As(III), indicating that the adsorption of As on Fe-NN/BFs was due to the iron needles. The adsorption of As(III) and As(V) on Fe-NN/BFs occurred very rapidly, especially for As(V). Fe-NN/BF achieved an As removal capacity with maximum values for As(V) and As(III) of 93.94 and 70.22 mg/g-Fe, respectively.

Also, Cuong et al. [251] used the same approach to remove arsenic. A rice-husk-derived biochar (BRH) prepared by means of the pyrolysis method in a nitrogen atmosphere at 700 °C for 8 h was used and compared with the active biochar (BC active) obtained from the BC of rice husks and subsequently activated by manganese dioxide (MnO_2_). The porosity characteristics of the active biochar were significantly increased compared to those of the rice charcoal. More specifically, the specific surface area and the pore volume of the active BC were 81.73 m^2^/g and 0.13 cm^3^/g, while those of the BC were only 37.04 m^2^/g and 0.03 cm^3^/g, respectively. Another important aspect is that the active BC had an extremely high mesoporosity ratio of 90%. The arsenic removal efficiency of the active BC filter was 3.49 mg/g, which was much higher than that of the BC, which was equal to 0.04 mg/g.

### 5.4. Chemical Properties: Influence on the Removal Efficiency

Chemical properties such as elemental composition, functional groups, bulk polarity, and ash content can affect the adsorption capacity of biochar. Abundant oxygen-containing groups are present on the surface of biochar (i.e., carboxylate, single COOH bonds, hydroxyl, and single OH bonds), which may have strong interactions with heavy metals, such as electrostatic attraction, ion exchange, and surface complexation [264]. Table 14 shows the effects of the chemical properties of biochar on heavy metals, including maximum adsorption capacity (q_m_) and removal efficiency (R).

An example can be encountered in the study by Xue et al. [94], which shows the results of the application of the following metals to a monometallic system with Pb(II) and to a multimetallic system: Ni(II), Cd(II), Cu(II), Pb(II). The biochar considered for adsorption was hydrothermally prepared from peanut shells (PHHC) and treated with hydrogen peroxide to obtain a modified biochar (mPHHC). The analysis of CHN revealed similar hydrogen contents between the two hydrochars; however, the carbon content of mPHHC (i.e., C = 48%) was lower than that of PHHC (i.e., C = 56%), while the oxygen content increased in mPHHC (i.e., O = 44%) compared to PHHC (i.e., O = 37%). Characterisation measurements showed that the H_2_O_2_ modification increased the number of oxygen-containing functional groups, especially the carboxyl groups, on the hydrochar surfaces. As a result, the modified hydrochar showed a greater adsorption capacity for Pb with a maximum capacity of 22.82 mg/g compared to PHHC, which was 1.04 mg/g. The removal capacity of mPHHC in the multimetal system followed the order Pb(II) > Cu(II) > Cd(II) > Ni(II). mPHHC was particularly effective in removing aqueous lead in both single and multimetal systems.

In the report by Chao et al. [249], the removal of metal ions of Cu(II), Cd(II), Ni(II) and Pb(II) was analysed using three types of agricultural wastes, Citrus maxima peels (CM), passion fruit peels (PF), and sugarcane bagasse (SB), to produce biosorbents. The materials were placed in a beaker in a 50 °C water bath for 48 h and then dried in an oven at 50 °C for 24 h. The CEC values of CM, SB, and PF are, respectively, 47.3, 11.8, and 26.9 m_eq_/100 g. The results show that ion exchange is one of the adsorption mechanisms for metal ions in the tested biosorbents. The CEC values of the biosorbents are derived from the functional groups present on their surfaces since functional groups such as carboxyl, amino, and hydroxyl provide complexation sites for metal ions on the surface of the biosorbents. CM showed the largest CEC values and the highest oxygen content, indicating that it has the highest ion exchange capacity among the three bioadsorbent materials. According to the CEC values, the maximum adsorption capacities of the biosorbents for divalent metal ions ranged from 173.0 to 70.2 mg/g. Adsorption capacities based on q_m_ are classified in the following order: Cu > Cd = Ni > Pb.

In the report by Abdallah et al. [146], biochar from spent mushroom compost (SMCB) was used. The material was dried at 100 °C for 24 h and then charred at a temperature of 500 °C for 3 h under limited oxygen conditions in a laboratory chamber oven. The characterisation of the material had shown an ash content (A) and a volatile matter value (VM) of 50.6 and 13.8%, respectively. The elemental components were C = 35.94%, H = 1.21%, N = 2.18%, and O = 38.83%, the CEC was 57.6 mmol/kg, and it had a pH of 8.8. The removal of heavy metals such as zinc, copper, and lead was also observed, and the final data show that the highest adsorption capacity of SMCB was with Pb(II) (i.e., q_max_ = 12.7 × 10^4^ mg/g), followed by Cu(II) (i.e., q_max_ = 1.11 × 10^4^ mg/g) and Zn (i.e., q_max_ = 0.60 × 10^4^ mg/g).

Fan et al. [265] studied the adsorption process using biochar obtained from the sewage sludge of a municipal water treatment plant (SDBC) to remove chromium. The biochar was activated by liquid phase impregnation with nano-zero-valent iron (nZVI-BC). Part of the pore structure of SDBC was blocked by nZVI particles after modification, and the surface iron content of nZVI-BC was much higher than that of SDBC. The surface functional groups of SDBC appear to be -OH, aromatic C=C, -COOH/-COOF-, aromatic C-H and F-O/Si-O-F. After the adsorption of Cr(VI), the peaks of nZVI and iron oxides were significantly reduced, indicating that there were chemical reactions between nZVI-BC and Cr(V). The result shows that the Cr species immobilised in the nZVI surface are mainly in the form of the less toxic Cr(III), which accounts for 82% of the total Cr. The dynamic performance of nZVI-BC in fixed-bed columns gave adsorption capacity values between 23.5 and 39.8 mg/g under different operating conditions.

Imran et al. [266] used biochar from *Chenopodium quinoa* (QBC) crop residues, pyrolysed at a temperature of 400 °C for 1 h at 8 °C/min, for the adsorption of Cr(IV). The results of biochar activated with magnetite nanoparticles (QBC/MNP) and biochar activated with strong acid HNO_3_ (QBC/Acid) were also observed. EDX analysis showed that QBC and QBC/Acid contained carbon and oxygen, while QBC/MNP also had iron on the surface of the quinoa biochar. The FTIR results confirmed the successful modification of QBC with MNP and acid. The results showed that QBC/MNPs proved to be more effective (i.e., R = 73.35–93.62%) for the removal of Cr(VI) with an adsorption capacity of 77.35 mg/g compared to QBC/acid (i.e., R = 55.85–79.8%) and QBC (i.e., R = 48.85–75.28%) when the Cr concentration was changed from 200 to 25 mg/L.

### 5.5. Experimental Apparatus and Operational Condition of Column System Studies

In the experiments on the removal of heavy metals by means of the use of biochar, the adsorption capacity is derived using the structures of the experimental set up, as previously shown. The dimensions of the column must ensure an easy sampling process and the hypothesis previously mentioned.

#### 5.5.1. Characteristics of Column, Layer, and Flow Used in the Literature

Experiments at the laboratory scale can be conducted using experimental columns with varying characteristics, including the material, the height (h), the diameter (**ϕ**), and operation parameters, among which are flow rate (Q) and the depth of the adsorbent bed (z), as shown in Table 15.

Column characteristics, i.e., height (h) and diameter (ϕ), should be suitable according to the physical characteristics of the material to be introduced. In fact, small columns can avoid the formation of preferential paths during the tests, but within these, the by-pass phenomena can occur on the walls of the column [267].

Table 16 summarises some configurations of the experimental equipment used (layers and operating conditions) and the relative removal efficiencies (R) and maximum adsorption capacities (q_m_) found.

Chao et al. [249] performed an adsorption experiment with only a single metal in the test to avoid competitive adsorption with RT being controlled at 25 °C. The effects of flow rate on adsorption capacity were investigated using experimental flow rates of 2.0, 3.0, and 4.0 mL/min. All experiments were performed in duplicate, and the quantitative results were averaged. The inner diameter of the fixed-bed column was 1 cm, and the length was 10 cm. The biosorbent formed a layer about 3.0 cm thick in the column. During the experiments, the water surface in the column was kept 2.0 cm above the biosorbent. As expected, it was observed that the adsorption capacities decreased with the increasing flow rate. The largest adsorption capacities were found with CM biochar for all four pollutants and all flow rates.

In another experiment conducted by Jellali et al. [250], the column was charged with a synthetic Pb solution at a specified initial concentration from the base of the column (upflow) using a fixed flow rate by means of a peristaltic pump. A plexiglass column was used with an inner diameter, length, and cross-section, respectively, of 2.9 cm, 20 cm and 6.6 cm^2^. For each experiment, a separate column was filled evenly with tiny amounts of cypress charcoal pre-treated with magnesium (Mg-B). Glass particles were placed at the edges of the columns to ensure uniform flow through the adsorbents used and water drainage. The amount of adsorbed lead increased with the decreasing flow rate. q_m_ was quantified at about 7.5, 7.9, and 8.8 mg/g for RCS and 85.5, 97.1, and 120.7 mg/g for Mg-B at the respective flow rates of 79, 59 and 45 mL/min. The mass of adsorbed lead increased when the depth of the adsorbent bed (z) increased.

In Tabassum et al. [93], the column-scale bio adsorption experiments were conducted in steady-state conditions using two peristaltic pumps: one on the inlet side and the other on the outlet side of the columns operating at the same flow rate. From the inlet side, As in solution was pumped through the capillaries (inner diameter: 2.5 mm) from a container, while the pump on the outlet side transferred the final product from the columns to the drain bottles. Five Plexiglas columns (i.e., h = 14.5 cm and ϕ = 4.5 cm) were used and were available in duplicate. Four columns were filled with two layers of fine, saturated sand (4 cm at the top and at the bottom) and a layer of gravel at the bottom (2 cm) and at the top (1.5 cm). The biochar was 3 cm thick and was placed in the centre. A fabric filter was placed at the top inlet and bottom outlet. Different biosorbents from agricultural organic waste and biochar were used to evaluate the removal efficiency using As-containing solutions and groundwater contaminated with As. After two hours, all biosorbents and biochar had removed 100% of the As from the aqueous solutions (i.e., C_0_ = 10 and 50 µg/L). After the following hour, all of the arsenic present in the contaminated water was removed at a higher initial concentration (i.e., C_0_ = 100 µg/L).

In another study by Wei et al. [96], RT column experiments were performed with Fe-NN/BF in packed-bed columns. A peristaltic pump was used to maintain an upward flow in the column. Fixed-bed columns with a diameter of 12 mm and a length of 100 mm were used. Approximately 10 mm thick quartz wool was used at each end of the column to distribute the flow, and the effective bed volume of the column was 9.04 mL. A satisfactory recycling performance of Fe-NN/BF could reduce the operating cost. Due to the highly hydrophilic nature and cross-linked pore structure of the Fe-NN/BF packed column, the column exhibits good hydraulic properties during long-term application. The packed Fe-NN/BFs column can be easily regenerated and reused many times. The adsorption capacity of biochar after chemical treatment showed good values of 93 mg/g for As(V) and 70 mg/g for As(III).

Cuong et al. [251] used a hybrid system achieved by coupling the fixed-bed column with electro-adsorption by capacitive deionisation (CDI) as a post-treatment to improve arsenic removal. A predetermined amount of material was packed wet into the packed bed to ensure good liquid distribution. A peristaltic pump was used to control the flow rates of influent and effluent in the column with an ascending flow pattern. The solution in the influent reservoir was continuously pumped into the column while the CDI was not operating. Subsequently, the solution flowing through the column was collected in an intermediate tank. After all of the solution in the influent tank was treated by the column adsorption experiment, the CDI cell was run with AC electrodes as a post-treatment step to deionise the solution in the intermediate tank. To control the arsenic removal capacity, CDI cells were assembled with different numbers of AC electrode pairs. The column used was made of glass with a length of 30 cm and an inner diameter of 2.7 cm. Inside, about 3 cm of acid-washed silica sand (average size: 0.5–0.6 mm) was filled at both ends, and a certain amount of biochar was filled in the middle. The integrated system showed a high potential for the removal of neutrally charged As(III). The adsorption capacity slightly decreases with the increasing flow rate.

#### 5.5.2. Effects of Variations of Biochar Dosage on Removal Efficiency

The dosage of the adsorbent, expressed by mass (m), mass per unit of volume (m/v), or mass per unit of weight (m/m_s_), has a significant influence on the adsorption efficiency; the use of an optimal biochar dosage (g, g/L or g/kg) for heavy metals removal is crucial for its cost-effective application [268].

The Table 17 below shows the variation in the adsorption capacity and removal efficiency as a function of the dosage variations.

In the column study conducted by Jellali et al. [250], the adsorption of lead was observed. A total of three adsorbent bed heights (2.6, 5.1, and 7.0 cm) were used, these heights corresponded to masses equal to, respectively, 5.0, 10.0, and 13.7 g. For biochar made from cypress sawdust (RCS) and biochar made from cypress sawdust activated with magnetised Mg particles (RCS-Mg-B), the removal efficiency increased with the depth of the adsorbent bed (z). q_m_ was quantified approximately at 6.9, 7.9, and 8.4 mg/g for RCS and 94.5, 97.1, and 97.0 mg/g for Mg-B for the beds used with heights, respectively, of 2.6, 5.1, and 7.0 cm.

In another experiment carried out by Choppala et al. [269], the contamination of As and Cr was investigated. Artificially contaminated soil and already contaminated soil resulting from the historical use of herbicides and the disposal of tannery sludge were studied. About 500 g of soil was treated with biochar made from chicken manure (BCM) (i.e., m = 50 g). In the red sandy clay soil, the addition of BCM reduced the concentration of Cr(VI) by 87.5% after 28 days of incubation; conversely, BC reduced the concentration of As(V) by 32.7%. In naturally contaminated tannery soils, Cr(VI) is reduced by 77.3% after 28 days of incubation with the same BC dosage. The reduction of Cr(VI) occurs more readily in soils in the presence of electron donors such as organic material. Conversely, the reduction of As(V) was slow even in highly reducing environments. The addition of BC increased the rate of reduction of As(V) by 1.2–2.3 fold in natural and field-contaminated soils. The effect was most pronounced for Cr(VI), which was increased by 2.5 to 4.0 times.

Naeem et al. [270] conducted laboratory-scale column experiments in which they applied about 5 g of biochar made from wheat straw (WSB) and biochar made from wheat straw acidified with H_3_PO_4_ (AWSB) with concentrations ranging from 0.5 to 8.0 g/L. The results of these experiments showed that the maximum Cd concentration (i.e., C_0_ = 100 mg/L) was adsorbed at 0.5 g/L of WSB and AWSB. The adsorption of Cd on biochar decreased with the increasing WSB and AWSB dosage. The highest Cd adsorption on AWSB was 60 mg/g, while on WSB, it was 40 mg/g at an 0.5 g/L dosage. The removal increased from 61 to 86% for AWSB and from 35 to 62% for WSB. The results showed that increasing the dosage of WSB and AWSB improved the efficiency of Cd removal, but there was a decrease in the Cd absorption capacity. This decrease in Cd adsorption could be due to the incomplete utilisation of the adsorption sites or to the formation of aggregates in response to a high dose of adsorbent.

In Zhang et al. [271], two treatments were established: no addition of biochar and the addition of 4% biochar made from corn straw (BCS) to the soil in the top layer of the column. The migration of heavy metals was affected by both the addition of DOM (dissolved organic matter) and biochar. The results showed that the release of DOM into the biochar-added medium resulted in a maximum removal of 57.5%, which was more than in the biochar-free medium, after 90 days. The Cd and Ni removal efficiency in the soil increased with the addition of biochar, from 16.2% to 28.7% and from 22.2% to 57.2%, respectively.

#### 5.5.3. Characteristics of the Inlet Contaminated Water

In Table 18 below, the characteristics and initial concentration (C_0_) of inlet waters used in some lab tests are presented.

#### 5.5.4. Contaminants Concentration Variation

Depending on the initial concentrations of the contaminants, it is possible to observe how adsorption capacity [273] is influenced.

Table 19 highlights the correlation between the initial concentration of contaminated water and the relative removal efficiency and adsorption capacity.

In Jellali et al. [250], lead nitrate (Pb(NO_3_)_2_) was used as a source of Pb ions. A lead aqueous solution of 1000 mg/L was prepared with distilled water and used in this study, and different initial Pb(II) concentrations (i.e., C_0_ = 25, 50, and 100 mg/L) were used. The results of the column experiments showed that for both cypress wood biochar (RCS) and cypress wood biochar magnetised with Mg (Mg-RCS), the efficiency of the adsorption process increases with the increasing initial Pb concentration. The experimental lead adsorption capacity is about 7.4, 7.8, and 7.9 mg/g for RCS and 77.1, 81.9, and 97.2 mg/g for Mg-B at initial Pb(II) concentrations of 25, 50, and 100 mg/L, respectively.

Bombuwala Dewage et al. [274] provided another example of Pb removal research. An aqueous Pb stock solution of 1000 mg/L was prepared by dissolving Pb(NO_3_)_2_ in deionised water (DI). The adsorption process was studied using a biochar made from pine wood and modified with chitosan (CMBC). Column studies were observed with an initial Pb concentration of 150 mg/L, reaching a maximum adsorption capacity of 5.8 mg/g.

In Tabassum et al. [93], aqueous solutions contaminated with As were prepared in a laboratory starting from a stock As solution (1000 mg/L) and dissolving an analytical-grade sodium arsenate salt (Na_2_HAsO_4_ + 7H_2_O) in distilled water. The solutions used had initial As concentrations of 10, 50, and 100 mg/L. In addition, six other As-contaminated groundwater samples were selected from Hasilpur (South Punjab, Pakistan). As concentrations in these groundwater samples were about 5, 10, and 50 mg/L. The arsenic removal potential of the biosorbents varied depending on the type, As concentration, contact time, and type of As solution. After one hour, the arsenic removal efficiencies of all biosorbents were 100, 100, and 90%, respectively, for 5, 10, and 50 mg/L As-contaminated groundwater samples, respectively, and 50%, 90%, and 90% for 10, 50, and 100 mg/L As solutions, respectively. After 2 h, all biosorbents and biochar had removed 100% of the As from the aqueous solutions, except for the 100 mg/L As a solution.

Also, in Cuong et al. [251], two aqueous solutions contaminated with As were prepared in a laboratory: one with NaAsO_2_ for As(III) and the other with As_2_O_5_ for As(V). A rice husk biochar activated with MnO_2_ (BC active) was used as an adsorbent in the column system, and different values of initial As concentrations (III) of 1.2 and 5 mg/L were used for the experiment. The maximum adsorption of arsenic increased significantly from 2.88 mg/g to 3.49 mg/g when the initial concentration increased from 1.0 to 5.0 mg/L. This can be attributed to the fact that a higher concentration gradient causes an increase in the driving force for the transport of arsenic from the solution to the active B.

Naeem et al. [270] used analytical-grade cadmium nitrate (Cd (NO_3_)_2_) to prepare a stock solution of Cd (i.e., C = 1.0 g/L) in distilled water. Experiments were performed with three different initial Cd concentrations (i.e., C_0_ = 25, 50, and 100 mg/L), and the Cd-contaminated water was continuously injected into a laboratory-scale column. In the first 90 min, the Cd removal rate changed from 89.2 to 93.35% and 92.3 to 95.6% using biochar made from wheat straw (WSB) and biochar made from wheat straw acidified with H_3_PO_4_ (AWSB), respectively, when the Cd concentration ranged from 100 to 25 mg/L. While after 240 min, WSB showed 71.6–77.5% removal, 78.4–86.28% of the Cd was removed from AWSB when the initial Cd concentration ranged from 25 to 100 mg/L. However, there was a decrease in the Cd removal rate with an increase in initial Cd concentration at each time. The difference in Cd removal between 25 and 50 mg/L is less than the Cd removal between 50 and 100 mg/L. This maximum Cd removal by WSB and AWSB could be attributed to the dosage and higher potential of WSB and AWSB due to the presence of different functional groups.

Another example of a study in which Cd removal was analysed can be found in Zhang et al. [275], in which a modified MgCl_2_ biochar from crab shell waste (MgC600) was used, and an initial concentration of Cd in the column that varied with values of 50, 100 and 150 mg/L was used. The flow rate was kept constant (i.e., Q = 5 mL/min). Due to the increased driving force of mass transfer, when the initial concentration of Cd (Ⅱ) is increased from 50 to 150 mg/L, the adsorption sites on the biochar surface are occupied more quickly, resulting in a decrease in the elapsed time. The adsorption capacities were 8.14 mg/g for 50 mg/L, 7.73 mg/g for 100 mg/L, and 8.44 for 150 mg/L.

In Imran et al. [266], the column scale study was performed at two different initial concentrations of Cr(VI): 50 and 100 mg/L. The column scale data showed excellent Cr retention during the 5 h injection at 50 mg/L, with maximum retention using magnetised quinoa biochar (QBC/MNPs), followed by acidified quinoa biochar (QBC/Acid) and quinoa biochar (QBC); however, a decrease in chromium removal via adsorbent was observed when 100 mg/L Cr was injected. The results showed that QBC/MNPs proved to be more effective (i.e., R = 86–78%) for the removal of Cr(VI) than QBC/Acid (i.e., R = 75–70%) and QBC (i.e., R = 61–54%) when the concentration of Cr was changed from 50 to 100 mg/L.

This influence can also be observed in Chen et al. [276], in which Cr-contaminated soil collected at a depth of 0–30 cm from a disused electroplating plant in Fujian Province, China, was examined. The behaviour of contaminated groundwater prepared by leaching deionised water from contaminated soil using a PVC column was also studied. In the contaminated soil, the Cr(III) concentration was 628 mg/kg, and the Cr(VI) concentration was 6622 mg/kg. In contrast, in the contaminated groundwater, the Cr(III) concentration was 126 mg/L, and the Cr(VI) concentration was 701 mg/L. The results showed that soil modified with biochar from barley grass and magnetised with Fe (Fe-BC) could remove about 71% of the Cr from contaminated groundwater. In contrast, different removal efficiencies were found for Cr(III) and Cr(VI) in the contaminated soils (78% and 22%, respectively). In the study, it was shown that simultaneous remediation to stabilise Cr in soil and remove Cr from groundwater gave satisfactory results.

#### 5.5.5. Effects of pH

Table 20 highlights the influence of pH on the efficiency of the removal of heavy metals from contaminated water and the related adsorption capacity.

The pH of the solution is one of the most vital parameters in optimising the adsorption process. The pH influence on adsorption may depend on the types of biochar and the target contaminants. It affects not only the surface charge of the adsorbent but also the degree of ionisation and the speciation of the adsorbate [231].

This effect can be seen in Chao et al. [249], in which three types of agricultural wastes were used to produce biosorbents for the removal of various metal ions. To investigate the effects of pH on adsorption capacity, the solutions were evaluated at pH 4.0, 5.0, and 6.0. The largest adsorption capacity was obtained with CM biochar for all four contaminants and all pH values. The results show a decreasing trend in adsorption capacity (Pb > Cd > Cu > Ni) according to obtained values (i.e., q_m_ = 169, 132, 84.0, and 60.7, respectively, for Pb, Cd, Cu, and Ni). In fact, at a low pH, the surface of the adsorbent was surrounded by hydrogen ions, which occupied the adsorption sites and reduced the adsorption capacity. Alternatively, a reduced adsorption capacity at low pH could be due to the limited complexation of metal ions with the biosorbents.

Another example can be found in Abdallah et al. [146], in which biochar obtained from spent mushroom compost (SMCB) with a pH of 8.8 was used. The experiment consisted of the removal of Zn, Cu, and Pb from an aqueous solution. The adsorption capacities were 0.60 mg/g for Zn, 1.11 mg/g for Cu, and 12.7 mg/g for Pb.

In Jellali et al. [250], the lead removal rate from aqueous solutions by crude cypress sawdust (RCS) and its pre-treated magnesium derivative biochar (Mg-B) was investigated under dynamic conditions through a column system. The pH of RCS was 5.7 and increased to 9.7 due to Mg-B after activation. The increase in pH also resulted in an improvement in the adsorption capacity; RCS biochar had values ranging from 6.9 to 8.4 mg/g, while those of Mg-B ranged from 77.1 to 97.2 mg/g, considering different operating conditions.

Xue et al. [94] used biochar hydrothermally prepared from peanut shells (PHHC) and activated with H_2_O_2_ (mPHHC). The untreated biochar had a pH of 6.2, while the pH of the activated biochar was 4.4. It can be observed that in the monometallic system for the removal of Pb, the adsorption capacity of the mPHHC was about 20 times higher than that of the PHHC, and respectively, they had a q_m_ of 22.82 and 1.04 mg/g. In contrast to the previous study, it is found that biochar with a lower pH has a higher adsorption rate.

Naeem et al. [270] investigated novel wheat straw biochar (WSB) and acid-treated wheat straw biochar (AWSB) for the removal of cadmium from contaminated water. Its adsorption was affected by several factors such as pH. The results showed that the difference between WSB and AWSB in Cd removal efficiency was the same even after changing the pH of the solution. The adsorption of Cd decreased with the decreasing pH, and the lowest adsorption was found at pH 2. The maximum Cd was removed at a pH of 6 with both the WSB and AWSB; therefore, the results showed a Cd removal efficiency of 35 to 82% with a capacity of 16 to 41 mg/g when the pH ranged from 2 to 6 for the WSB, while for the AWSB, the efficiency was higher from 45 to 98% and with a capacity of 23 to 48 mg/g.

## 6. Analytical and Numerical Models for the Interpretation of the Results

There are several analytical and numerical models which can be used to represent the adsorption process in the column experiments. Below, the most known models are briefly summarised.

### 6.1. Analytical Models

#### 6.1.1. Bohart–Adams

Bohart–Adams model (BA) assumes that the adsorption rate can be described using an irreversible first-order kinetics concerning both the C_0_ concentration of the absorbable component present in the fluid and the residual adsorption capacity of the adsorbent material per unit of bed volume [247]; therefore, it assumes that the equilibrium is not instantaneous, and that the adsorption rate is proportional to the remaining adsorption capacity on the adsorbent. The linearised form of the model is as follows [277]:

(1) $$\ln\left(\frac{C}{C_{0}}\right)=K_{BA}C_{0}t-K_{BA}\frac{\;q_{m}z}{u}$$

where *t* is the time (min), *C*_0_ is the concentration entering the column (mg/L), *C* is the output concentration (mg/L), *z* is the depth of the adsorbent bed (cm), *u* is the linear velocity of influent solution (cm/min), *K_BA_* is the Bohart–Adams rate constant [L/(mg min)], and *q_m_* is the maximum adsorption capacity (mg/g). Both *K_BA_* and *u* can be determined from the slope and intercept of the line obtained by plotting the variables of the model’s linearised equation. The parameters of the Bohart–Adams Equation (1) can be obtained experimentally and used for the sizing of an adsorption column. For example, by setting *t* = 0 and the output concentration equal to *C*, the minimum required value for the height of the column can be determined [278].

#### 6.1.2. Thomas

Thomas’s model is frequently adopted to predict the breakthrough adsorption curve in a fixed-bed model [249,279]. The linearised form of Thomas’ model is as follows [280]:

(2) $$ln\left(\frac{C_{0}}{C}-1\right)=\frac{k_{Th}q_{m}m}{Q}-k_{Th}C_{0}t$$

where *C*_0_ is the concentration entering the column (mg/L), *C* is the concentration leaving the column at time *t* (mg/L), *t* is the time (min), *m* is the mass of adsorbent (g), *Q* is the flow rate (mL/min), *k_Th_* [mL/(min mg)] is the constant of the Thomas model, and *q_m_* (mg/g) is the expected maximum adsorption capacity. 

Thomas’s model is based on the assumptions of Langmuir adsorption–desorption kinetics and no axial dispersion. Using Equation (2), we can estimate the maximum adsorption capacity of metal ions under the given conditions. Axial diffusion is not considered in the model; therefore, variations in the flow rate can determine substantial differences in the adsorption capacity [281].

#### 6.1.3. Modified Dose Response

The modified dose-response model (MDR) is a simplified mathematical model capable of reducing the error derived from the Thomas model, particularly in periods below or above the breakthrough curve [282]. The model is described by Equation (3):

(3) $$ln\left(\frac{C}{C_{0}-C}\right)=a\;lnC_{0}Qt-a\;ln({m\;q}_{m})$$

where *t* is the breakthrough time (min), *C*_0_ is the input concentration (mM), *C* is the output concentration (mM), *m* is the mass of the adsorbent (g), *q_m_* is the maximum adsorption capacity (mg/g), and *a* is the constant of the modified dose-response model. Both *a* and *q* can be determined by the slope and intercept of the line obtained by plotting the variables of the model’s linearised equation [280].

#### 6.1.4. Yoon–Nelson

The Yoon and Nelson model (YN) is a fixed-bed adsorption kinetic model. This model can be considered simpler than other models. As reported in [277] it does not require detailed information on system characteristics like the type of adsorbent and/or the physical properties of the adsorbent bed. The Yoon–Nelson linearised model is represented by Equation (4):

(4) $$ln\left(\frac{C}{C_{0}-C}\right)=k_{YN}t-\tau k_{YN}$$

where *t* is the processing time (min), *C*_0_ is the concentration entering the column (mg/L), *C* is the output concentration (mg/L), *τ* is the time required for the passage of 50% of the adsorbate (min), and *k_YN_* is the constant of the Yoon–Nelson model (1/min) [283]. The values of *k_YN_* and *τ* are obtained from the slope and intercept of the linear graphs of the variables of the model’s linearised equation [278].

#### 6.1.5. Clark

The Clark model [284] was developed to study adsorption performance using Equation (5):

(5) $$ln\left[{\left(\frac{C_{0}}{C}\right)}^{n-1}-1\right]=-rt+lnB$$

where *C*_0_ is the input concentration (mg/L), *C* is the output concentration (mg/L), *t* is the breakthrough time (min), *n* is the Freundlich constant (-), and *r* (mg/L min) and *B* (min) are the Clark model constant.

This model assumes that the Freundlich isotherm is applied, i.e., there is a small initial increase in the quantity of adsorbed material as a function of the equilibrium concentration due to the existence of weak adsorption forces in correspondence with the first adsorbate layer, and that the adsorption rate is limited by the external mass transfer phase [146].

### 6.2. Numerical Models

#### 6.2.1. Advection Dispersion Equation

The advection dispersion equation (ADE) model is based on the balance of mass as shown in Equation (6), which describes the transport and dispersion of a solute in a saturated porous media:

(6) $$\frac{\partial C}{\partial t}+u_{i}\frac{\partial C}{\partial x_{i}}-\frac{\partial }{\partial x_{i}}\left(D_{ij}\frac{\partial C}{{\partial x}_{j}}\right)=0$$

where *C* is the solute concentration of the adsorbate within the column over time (mg/L), *t* the time (min), *D_i,j_* the hydrodynamic dispersion tensor (cm^2^/min), *x_i_* is the axes, and *u_i_* is the components of the velocity vector (cm/min) [285].

The integration of the advection–dispersion Equation (6) is usually performed numerically. In column tests, the experimental set-up can be approximated to a one-dimensional system, with the hydrodynamic dispersion coefficient and the hydraulic conductibility assumed to be constant (the hypothesis is surely valid in the case of a homogeneous filling material):

(7) $$\frac{\partial C}{\partial t}-u\frac{\partial C}{\partial x}=D\frac{\partial^{2}C}{\partial x^{2}}$$

where *u* is the only non-zero component of the fluid velocity (cm/min). One of the most used integration methods is the finite difference method.

The application of this method requires the definition of the initial and boundary conditions and the consideration of the effects of numerical diffusivity, which is associated with the presence of the convective transport term that, together with the stability problems of the solution, conditions the integration. Luciano et al. [286] provided a solution to this problem by introducing a method for computing the integration time step that, at least to a first approximation, solves both the numerical diffusivity and stability problems of the solution [285,287].

To consider the adsorption of the solute onto solid particles of porous media it is necessary to modify the advection–dispersion equation as follows:

(8) $$\frac{\rho_{b}}{p}\;\frac{\partial S}{\partial t}+\frac{1}{p}\frac{\partial C}{\partial t}=\frac{D}{p}\frac{\partial^{2}C}{\partial x^{2}}-\frac{u}{p}\frac{\partial C}{\partial x}$$

where *S* is the amount of solute absorbed on the porous media particles, *p* is the porosity (-), and *ρ_b_* is the bulk density (mg/cm^3^).

#### 6.2.2. CXTFIT

This model was used to solve the advection–dispersion equation without the adsorption, volatilisation, or biodegradation phenomena and assumes that the effect of diffusion on the effective dispersion coefficient can be ignored. It was used to simulate the transport of the conservative tracer to examine any water in the columns. The adsorption parameters obtained by the CXTFIT program correspond to the K_d_ values of the linear isotherm; however, adsorption is generally not linear for heavy metal ions [288]. Equation (9) below describes the model:

(9) $$\frac{\partial C}{\partial t}=-u\frac{\partial C}{\partial x}+\lambda u\frac{\partial^{2}C}{\partial x^{2}}$$

where *C* is the concentration of the adsorbate within the column over time (mg/L), *t* is time (s), *x* is the direction of motion (or axial dispersion), *u* is the interstitial water velocity (cm/s), and *λ* is the longitudinal dispersion coefficient (-) [250].

#### 6.2.3. Hydrus 1D

The Hydrus 1D model is a numerical model that uses the finite element method to solve the flow equation and the advection–dispersion equation, and unlike the CXTFIT model, it can predict the breakthrough curves, assuming nonlinear adsorption and also reactive contaminants in the presence of non-equilibrium physical and/or chemical processes [289,290]. The 1D vertical transport of a non-volatile and non-biodegradable solute in saturated or unsaturated porous media can be described by the advection–dispersion Equation (10), as follows:

(10) $$\theta \frac{\partial C}{\partial t}+\rho \frac{\partial S}{\partial t}=-\theta u\frac{\partial C}{\partial x}+\theta \lambda u\frac{\partial^{2}C}{\partial x^{2}}$$

where *C* is the concentration of the adsorbate within the column versus time (mg/L), *t* is time (s), *u* is the velocity of interstitial water (cm/s), *x* is the direction of motion, *λ* is the longitudinal dispersion coefficient, *θ* is the volumetric water content (cm^3^/cm^3^), *S* is the ratio between the solute concentration in the solid and aqueous phases, *ρ* is the average mass density (g/cm^3^) [291], and the parameter *S* is given by the following empirical isotherm:

(11) $$S=K_{d}\frac{C^{\beta }}{1+\mu C^{\beta }}$$

where *K_d_* (cm^3^/g) is the absorption distribution coefficient, and *β* (-) and *μ* (cm^3^/g) are constants related to the shape of the isotherm [250]. 

Solute transport in porous media may be strongly affected by some chemical and/or physical non-equilibrium processes. The chemical non-equilibrium model (CNEM) corresponds to a kinetic behaviour that usually occurs when the interactions between the solute and the adsorbent are slow compared to the residence time [292].

Therefore, the total amount of contaminant (S) retained by the solid matrix can be divided into a fraction of contaminants immediately absorbed at the equilibrium sites (S_1_) and a fraction of contaminants kinetically absorbed at the first-order kinetic sites (S_2_), as described by the following two Equations (12) and (13):

(12) $$S_{1}=K_{d}\frac{C^{\beta }}{1+\mu C^{\beta }}$$

(13) $$\frac{{\partial S}_{1}}{\partial t}=\alpha \left[\left(1-F_{c}\right)\;\frac{K_{d}C^{\beta }}{1+\mu C^{\beta }}-S_{2}\;\right]$$

where *S*_1_ and *S*_2_ are the solute concentrations in the solid phase for the first and second adsorption sites, respectively; *F_c_* (-) is the fraction of sites that are in instantaneous equilibrium; and *α* is the first-order rate coefficient (1/min) [293].

Hydrus 1D uses an inverse modelling function based on the nonlinear Levenberg–Marquardt least squares optimisation algorithm to find the model input parameters that best fit the experimental data [294].

### 6.3. Analytical and Numerical Model Calibration

Table 21 highlights the values of some parameters obtained using pseudo-first- (PFO) and -second-order (PSO) kinetic models. 

Adsorption kinetics studies are important because they provide information about the rate of the adsorption process and reveal the main factors affecting the reaction rate. Several kinetic models have been developed to study the different adsorption mechanisms [122].

For example, in Xue et al. [94], PFO, PSO, and Elovich models were used to simulate the adsorption kinetics data for the removal of Pb and Cd. Pb adsorption on biochar from peanut shells activated with H_2_O_2_ (mPHHC) occurred much faster and to a greater extent than on biochar from peanut shells (PHHC), suggesting that treatment with H_2_O_2_ may facilitate the adsorption of lead on hydrocarbons. It took less than 12 h for the adsorption of lead on mPHHC to reach the apparent equilibrium, while the absorption equilibrium on PHHC was reached even after 24 h. Moreover, the kinetic adsorption curve of mPHHC was smoother than that of PHHC, indicating less surface heterogeneity of mPHHC after H_2_O_2_ modification. The kinetic models described the experimental data quite well, with R^2^ being greater than 0.86. Elovich’s model gave the best results for both PHHC (i.e., R^2^ = 0.89) and mPHHC (i.e., R^2^ = 0.99), which were not much better than the second-order model (i.e., R^2^ = 0.89 and 0.96, respectively). The PFO model gave the worst results for both PHHC (i.e., R^2^ = 0.86) and mPHHC (i.e., R^2^ = 0.90).

In another study by Mahdi et al. [280], PFO and PSO kinetic models were applied to the experimental data for the removal of Pb. The adsorption rate reached equilibrium within 360 min and then became constant (24 h). The experimental data are better integrated by the PSO model, with R^2^ = 0.94, k_PSO_ = 3.007 × 10^−3^ [g/(mg min)], and q_e_ = 9.117 (mg/g), than by the PFO model, with an R^2^ = 0.86, k_PFO_ = 0.011 (1/min), and q_e_ = 9.117 (mg/g). This indicates that chemisorption was the dominant mechanism.

In Wei et al. [96], PFO and PSO kinetic models were used. Adsorption was well described by the PSO kinetic model, suggesting that the adsorption process could be chemisorption. A higher value at a constant rate for As(V) adsorption indicates that the biochar from cotton fibres (BF) magnetised with Fe (Fe-NN/BF), the removal effect was faster for As(V) than for As(III). Overall, 100% of the As(V) was removed within 10 min with a 2 g/L adsorbent and within 15 min with a 1 g/L adsorbent. When a 0.5 g/L adsorbent was used, 100% of the As(V) was removed in 120 min. In addition, the adsorption equilibrium of As(III) was achieved in about 60 min using a 2.0 g/L adsorbent and 240 min using a 0.5 g/L adsorbent. The rapid adsorption of As was attributed to the short diffusion distance and high accessibility of the adsorption sites.

Similarly, Cuong et al. [251] used nonlinear PFO and PSO models. The results indicate that biochar made from rice husks and activated by MnO_2_ (BC active) removes both As(III) and As(V) much better and faster than biochar made from rice husk (BC), which is probably due to the improved porous structure and abundant active sites of MnO_2_. Moreover, the PSO model fits the experimental data better with higher correlation coefficients (i.e., R^2^ > 99.2%) than the PFO model (i.e., R^2^ > 80.0%). This result confirms that the removal of As(III) and As(V) from BC and active BC is controlled by a chemical mechanism. The removal of As(III) and As(V) from BC reached equilibrium at approximately 120 min with removal capacities of 0.18 and 0.44 mg/g, respectively. The equilibrium times of active BC for the removal of As(III) and As(V) were about 60 min with removal capacities, respectively, of 1.36 and 2.12 mg/g.

The kinetic results of Cr removal by Imran et al. [266] showed a value of the determination coefficient (R^2^) for the PSO kinetic model of 0.99, which is larger than the R^2^ value obtained for PFO model. Thus, the experimental kinetic data were well explained by the PSO model. The calculated q_e_ values of the PSO model for quinoa biochar (QBC), quinoa biochar magnetised with Mn (QBC/acid), and quinoa biochar acidified with HNO_3_ (QBC/MNPs) were 16.37, 12.77, and 15.22 mg/g, respectively, while the corresponding values of the PFO kinetic model were 6.67, 5.29 and 5.38 mg/g.

It is also important to observe the parameters obtained from the models based on the interpretation of the breakthrough curves for the study of column systems. Table 22 highlights the values of the parameters of the related models that best fit the experimental data of the breakthrough curves.

The breakthrough curves are used to study the trend of the concentration of the adsorbable component in the effluent from the column [295,296]. The suitability of the kinetic model and of the equation used to describe the process of interaction between solute and porous media is verified by checking the output of the numerical/analytical model and the experimental data. A calibration phase can help to obtain a better link between the numerical and experimental data and to derive some of the unknown parameters.

**Figure 6:**
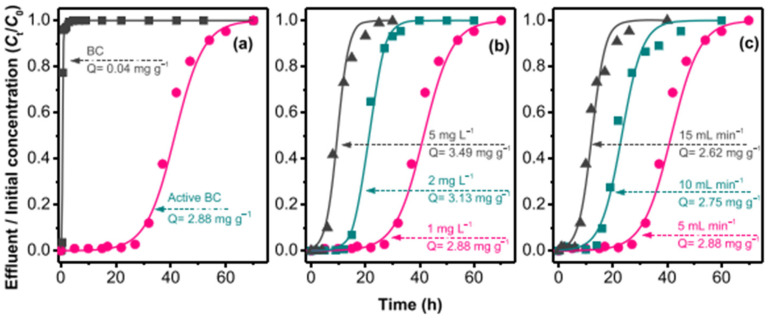
Measured breakthrough curves (points) adapted (lines) for active BC with a synthetic arsenic solution (**a**). Filtration experiments were also conducted under different (**b**) initial arsenic concentrations of 1, 2, and 5 mg/L and (**c**) flow rates of 5.10 and 15 mL/min [251].

An example of a study using Thomas’ model is reported in Cuong et al. [251], in which data were collected through experimental activities carried out also at different initial concentrations and flow rates. The adaptation of these data and the study of the model are shown in Figure 6.

In Figure 6a, it is possible to highlight that the maximum arsenic adsorption of active BC (2.88 mg/g) was much greater than that of BC (0.04 mg/g). The residence time of the BC active filter was 27 h, while that of BC was only 0.5 h. These results are due to the larger number of active sites on active BC. At As concentrations of 1, 2, and 5 mg/L, Figure 6b, the breakthrough times were 27, 12 and 4 h, respectively. The maximum adsorption of arsenic increased significantly from 2.88 mg/g to 3.49 mg/g, while the initial concentration increased from 1 to 5 mg/L. The adsorption rate of arsenic, k_Th_, decreased from 0.003 to 0.0016 [L/(mg min)] when the concentration was increased from 1 to 5 mg/L. As the flow rate increased from 5 to 15 mL/min, Figure 6c, the maximum adsorption capacity of arsenic decreased from 2.88 to 2.62 mg/g, but k_Th_ increased from 0.0025 to 0.0057 [L/(mg min)]. In addition, the breakthrough time decreased from 27 to 6 h when the flow rate was increased from 5 to 15 mL/min. 

**Figure 7:**
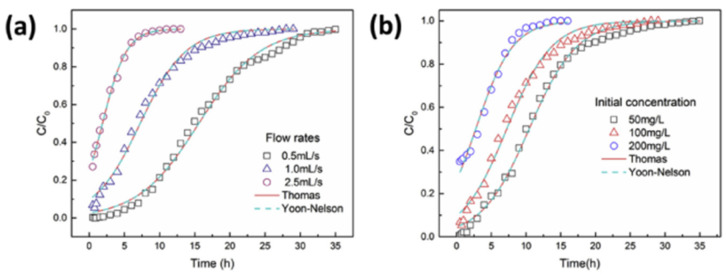
Cr(VI) breakthrough curves in nZVI-BC columns under various conditions: (**a**) effect of flow rates, and (**b**) effect of initial chromium concentration [265].

Fan et al. [265] used both the Thomas and Yoon–Nelson models, and the results are shown in Figure 7.

In Fan et al. [265], the simulated breakthrough curves of the two models, are almost identical, with R^2^ values above 0.99. In Thomas’ model, the best-fit value of k_Th_ increases with the increasing flow, while q_m_ shows a reverse trend. On the other hand, k_Th_ decreases with the increasing input concentration. In the Yoon–Nelson model, k_YN_ increases with the increasing flow rate and initial Cr(VI) concentration. The breakthrough curves were studied with different operating parameters such as the flow rates and initial concentration. At a slow flow rate (0.5 mL/s), the Cr(VI) anions in solution had more time to interact with the nZVI-BC bed and therefore exhibited a greater distance. Over a relatively long period (10 h), the filter maintained a removal efficiency of about 80%. Increasing the flow rate (2.5 mL/s) resulted in a lower removal of Cr(VI) and thus a shorter saturation time and steeper breakthrough curves. By contrast, an increased initial Cr(VI) concentration resulted in the faster saturation of the nZVI-BC sorbent in the fixed bed. Consequently, Cr(VI) showed a faster reversal at a higher initial concentration.

**Figure 8:**
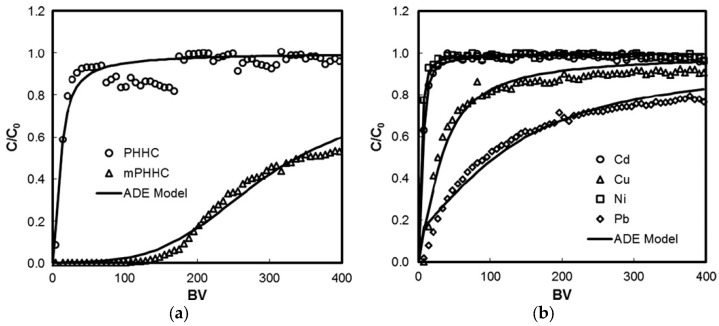
Removal and transport, respectively, of heavy metals in hydrochar columns: (**a**) Pb, and (**b**) Cd, Cu, Ni, and Pb [94].

Another example of the use of breakthrough curves is from the study by Xue et al. [94], in which the filtration and transport of heavy metals in hydrocarbon columns were described by the advection–dispersion equation (ADE) coupled with an Elovich equation modified to describe heavy metal removal and transport, as shown in Figure 8, in which BV and C/C_0_ represent the bed volume and relative concentration.

In the monometallic system, shown in Figure 8a, Pb breakthrough occurred much later in the mPHHC (modified biochar) column, and much lower lead concentrations were eluted than in the PHHC column. At the end of the experiment, the lead concentration in the final effluent in the PHHC column was close to the input concentration (i.e., C/C_0_ ∼ 1), while the mPHHC column still effectively removed about half of the lead in the influent. The ADE model simulated the lead breakthrough curve data well, and the q_max_ of the PHHC column was estimated to be about 1.04 mg/g, which is slightly larger than that of the batch column but still much smaller than that of the mPHHC column.

In the multimetal system, shown in Figure 8b, the column lost the ability to remove Cd(II) or Ni(II) after about 40 BV, and the two breakthrough curves stabilised at C/C_0_ = 1. Although Pb(II) and Cu(II) in solution had the same molar concentration as Cd(II) or Ni(II), their removal by the mPHHC column was much larger. After 400 BV, the mPHHC column could still remove about 10% of the Cu(II) and more than 20% of the Pb(II) from the solution. The ADE model also described all the breakthrough data well in the multimetal experiment. The q_max_ values indicate that Pb(II) and Cu(II) competitively occupied most of the adsorption sites on the mPHHC (72.1 and 17.1%, respectively).

**Figure 9:**
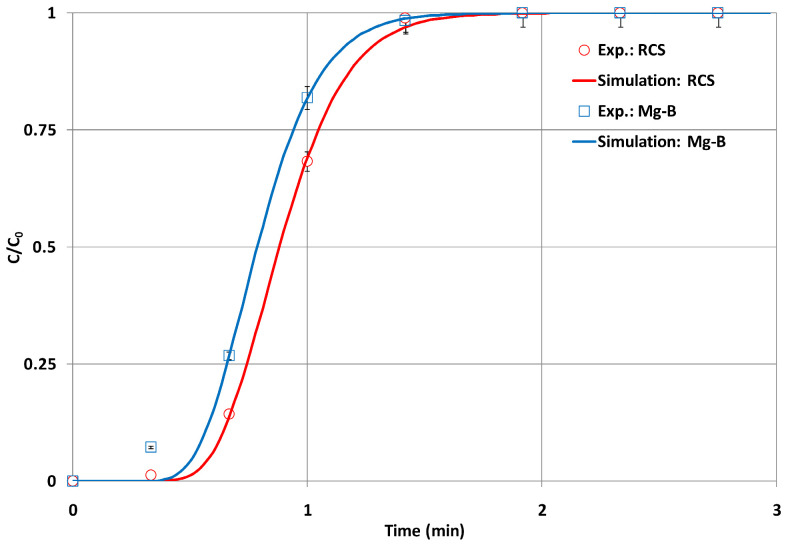
Observed and adapted breakthrough curves of the conservative tracer with the CXTFIT model for RCS and Mg-B [250].

In Jellali et al. [250], the CXTFIT and Hydrus 1D models were analysed. The results are shown in Figure 9.

Breakthrough curves for RCS and Mg-B were nearly symmetrical with no presence of tailing at the end (when C/C_0_ was close to 1). The best breakthrough curves were obtained for real velocity and longitudinal dispersion values, respectively, equal to 11.5 cm/min, 0.34 cm for RCS, and equal to 12.9 cm/min and 0.37 cm for RCS-Mg-B.

**Figure 10:**
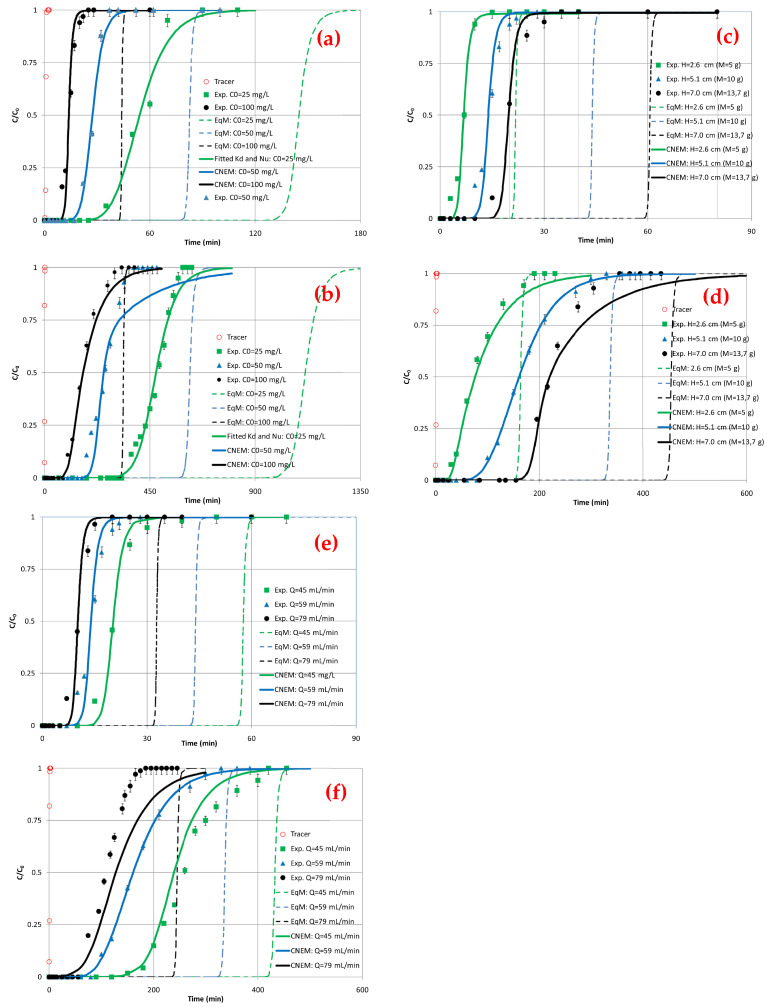
Breakthrough curves observed and adapted with equilibrium and chemical non-equilibrium models for RCS and Mg-B as presented by Jellali et al. [250]. Top-left graph: (**a**) for RCS and (**b**) for Mg-B, with different initial Pb concentration (C_0_ = 25, 50 and 100 mg/L); pH_0_ = 4.0, Q = 59 mL/min, z = 5.1 cm, T = 20 ± 2°C. Top-right graph: (**c**) for RCS and (**d**) for Mg-B, with different depths of the adsorbent bed (z = 2.6, 5.1 and 7.0 cm); pH_0_ = 4.0, C_0_ = 100 mg/L, Q = 59 mL/min, T = 20 ± 2°C. Bottom graphs: (**e**) for RCS and (**f**) for Mg-B, with different flow rates (Q = 45, 59 and 79 mL/min); pH_0_ = 4.0, C_0_ = 100 mg/L, z = 5.1 cm, T = 20 ± 2°C.

In the same study, the Hydrus 1D model was used to predict the lead breakthrough curves under different experimental conditions such as initial concentrations, feed rate, and the depth of the adsorbent bed. The main breakthrough curves (BTCs), shown in Figure 10, were simulated using the two-sided CNEM with Fc and α (first-order velocity coefficient) as fitted parameters.

The results showed that the predicted BTCs agreed very well with the experimental curves. The use of Hydrus 1D showed that the two-sided non-equilibrium chemical model can be used to adequately describe the experimental lead breakthrough curves for both RCS and Mg-B as equilibrium models, with coefficients of determination (R^2^) > 0.97 [250].

**Figure 11:**
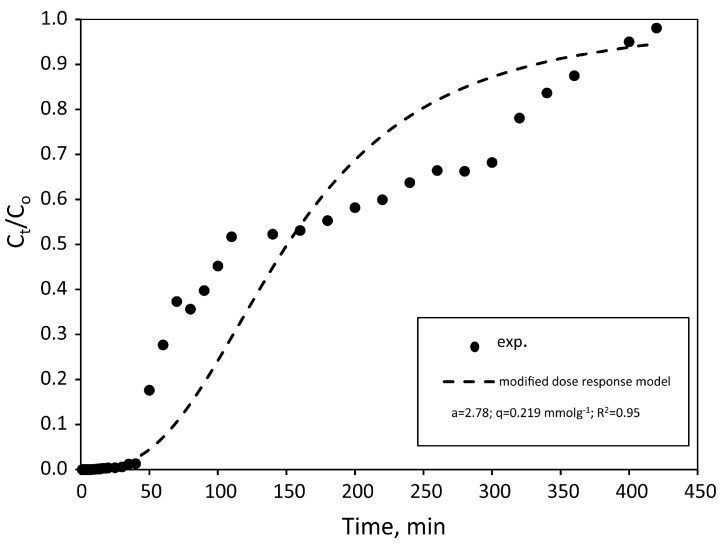
Breakthrough curve of Pb(II) adsorption, with z = 5.0 cm; Q = 1.0 mL/min; C_0_ = 0.5 mM; D = 0.6 mm; pH = 6.0 [280].

Mahdi et al. [280] used the Bohart–Adams model, the Thomas model, and modified dose-response models for the column study. Both the Bohart–Adams model (i.e., R^2^ = 0.76) and the Thomas model (i.e., R^2^ = 0.77) showed a reasonable fit to the experimental data; however, the experimental data for adsorption showed a better fit with the modified dose-response model (i.e., R^2^ = 0.95), as shown in Figure 11.

According to the modified dose-response model, the maximum adsorption capacity of Pb(II) in the packed column was found to be 4.538 × 10^2^ mg/g. The adsorption capacity for Pb(II) adsorption onto the biochar was much lower than the one obtained in the batch tests. This may be attributed to the insufficient contact time, which did not allow the Pb(II) to diffuse into the biochar particles, so the adsorption process may still have been ongoing and adsorption equilibrium had not yet been reached [280].

**Figure 12:**
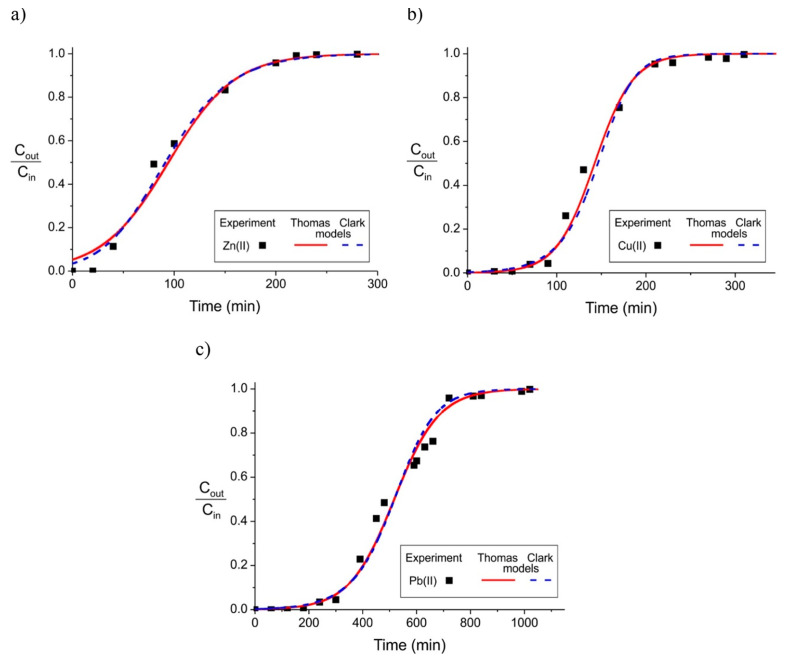
Breakthrough curves of in a single-metal system. (**a**) Zn, (**b**) Cu, and (**c**) Pb ions described by the Thomas and Clark models in a fixed-bed continuous adsorption [146].

Abdallah et al. [146] investigated the adsorption of Zn(II), Cu(II) e Pb(II) on SMCB using a fixed-bed continuous column. For each metal, the breakthrough curve was obtained by collecting samples of ion solutions at the outlet of the column, which are shown in Figure 12.

Thomas and Clark models were used to fit the curves, and the results showed a high correlation factor for both models: Pb(II) has the highest adsorption capacity (i.e., q = 12.70 × 10^4^ mg/g), followed by Cu(II) (i.e., q = 1.11 × 10^4^ mg/g) and Zn(II) (i.e., q = 0.60 × 10^4^ mg/g). Competitive adsorption was also studied by injecting a solution containing the three metals in the fixed-bed continuous column. The obtained results confirmed the higher affinity of Pb(II) to SMCB than Zn(II) and Cu(II) [146].

## 7. Conclusions and Future Perspectives

The aim of this review article was to report the state of the art on the removal of heavy metals from waters using biochar as an adsorbent material. This paper deals specifically with tests carried out at the lab scale using column systems in order to evaluate the possible real-scale application in groundwater remediation through, for example, a reactive permeable barrier (PBR).

It is possible to observe from these experiments that feedstock and the temperature of pyrolysis are the main factors that determine the physic-chemical properties of the biochar, in particular, heavy metal removal efficiency. From the several tests, it is possible to underline that physical, and chemically modified biochars generally present a higher adsorption capacity if compared to raw or untreated biochar. In particular, high removal efficiencies were observed when the biochar was treated with Mg or acidified with H_3_PO_4_ because the specific surface area increased after modification. 

Other aspects from the carried-out experiments is that the adsorption capacities decreased proportionally with the flow rates. Another important aspect is the biochar dosage. In fact, increasing the amount of adsorbent the adsorption capacities increase as expected. pH value variations can modify adsorption. In most cases, increasing the pH also increases the removal efficiency.

Regarding the kinetic models, PSO was the one that better fits with the experimental data, followed by PFO, suggesting the chemisorption nature of the adsorption process. The most used model is the Thomas model, but the Yoon–Nelson and Hydrus 1D models showed the highest coefficients of determination.

We hope that the effort to summarise the contributions from several researcher in one single work is useful for the future application of this studied material. The main challenge of using biochar on a large scale is finding out how to engineer its application at the field scale.

Columns in the pump-and-treat approach may certainly be employed, and lab-scale experiments can surely be used for calculating the column volume. Interest may arise in evaluating the application of this material using fixed beds or PRB, and in these cases, lab-scale studies are a useful source of information.

Therefore, future efforts must be directed toward the development of field-scale technologies that, based on lab-scale experiments, can introduce the use of biochar as an alternative material in the groundwater remediation field that also respects the concept of circularity.

## Figures and Tables

**Table 1:** Characterisation methods for biochar, adapted from Nartey and Zhao [46].

Analysis	Characterisation
Nitrogen Adsorption Isotherms	It is generally used to determine the surface area of biochar. It can be calculated based on the multilayer adsorption model of Brunauer–Emmett–Teller (BET). The surface area of biochar determines the multilayer adsorption capacity under a different partial pressure of nitrogen.
XRD (X-ray Diffraction)	It is used to analyse the carbon crystallites by determining the angle and intensity of diffracted beams.
FTIR (Fourier-Transform InfraRed spectroscopy)	Used to identify the chemical functional groups on the biochar. It is based on the waves in the frequency domain of light intensity using the Fourier transform function.
XPS (X-ray Photoelectron spectroscopy)	Used to define the valence state of chemical elements by analysing the energy distribution of photoelectrons.
SEM (Scanning Electron Microscopy)	Used to observe the surface morphologies by means the observation of the interaction between the electron beam and the samples.
TEM (Transmission Electron Microscopy)	Used to analyse the surface morphologies. It is possible to obtain high-resolution results.
EDS (X-ray Spectroscopy)	EDS is usually used together with an electron microscope. The combination allows for the determination of the molar fraction of the elements on the surface of biochar using the X-ray photon characteristic energy of each element.
STEM (Scanning Transmission Electron Microscopy)	It can characterise the biochar at the atomic scale.
EXAFS (X-ray Absorption Spectroscopy)	This technique can characterise the poly-aromatic structure of biochar according to the mechanism of multiple scattering resonances.
NMR (Nuclear Magnetic Resonance)	It can determine the carbon aromaticity and the content of protonated and non-protonated carbon in biochar by means of Zeeman splitting the spin level of the nucleus under an external magnetic field.

**Table 2:** Temperature, residence time, and yields (wt% based on dry feedstock) of the different pyrolysis processes, adapted from Qambrani et al. [82].

Process	Temperature (°C)	Residence Time	Yields (%)		
			Biochar	Bio-Oil	Syngas
Fast Pyrolysis	500–1000	<2 s	12	75	13
Intermediate Pyrolysis	~500	10–20 s	20	50	30
Slow Pyrolysis	300–700	hour-days	35	30	35

**Table 3:** Different feedstock and operating conditions for biochar production (producers and models are just a reference for the reader).

Feedstock Type	Process	Reactor	Model/ Producer	Residence Time (min)	Temperature (°C)	Heating Rate (°C/min)	References
Biowaste	Pyrolysis	Muffle furnace	BST-06 Beston group	20	350	8–10	[93]
Peanut hull	Hydrothermal carbonisation	Autoclave	Olten Environmental	300	300	n.d.	[94]
	Pyrolysis	Muffle furnace	n.d.	n.d.	300	n.d.	[95]
Cotton fibre	Pyrolysis	Tubular furnace	Anhui Kemi Instrument Co–TF series	120	800	n.d.	[96]
Dairy manure	Pyrolysis	Muffle Furnace	n.d.	240	350	25	[97,98]
Wood biomass	Pyrolysis	Pyroliser	Dinghi Group	n.d.	750	n.d.	[99]
Woody yard waste	Pyrolysis	Full-scale pyrolysis furnace	Beston/Mingjie	15 × 60	500	n.d.	[100]
Virgin coniferous wood	Pyro-gasification	Pyro-gasifier	Kj group	n.d.	600	n.d.	[101]
Beech woody biomass	Plant carbonisation	Mobile furnace	Mingjie Group	n.d.	400–800	n.d.	[102]
Oak, sycamore, birch, and cherry	Pyrolysis	Steel ring furnace	BNRTherm	n.d.	400	n.d.	[59]
Water hyacinth plants	Slow pyrolysis	Muffle furnace	n.d.	180	450	5	[103]
Hickory	Pyrolysis	Bench-top furnace	Beston/Sigma-Aldrich	120	600	20	[104]
Brewers draft	Pyrolysis	n.d.	n.d.	n.d.	650	5	[105]
Chicken bone	Slow pyrolysis	Furnace	Beston	240	600	10	[106]
Sewage sludge (SS)	Pyrolysis	Muffle furnace	n.d.	120	400	10	[107]

**Table 4:** Statistical summary of the characteristic of biochar, modified from Ahmad et al. [111].

Statistical Parameters	Temperature (°C)	Heating Rate (°C/min)	η (%)	A (%)	pH	C (%)	H (%)	O (%)	N (%)	S_BET_ (m^2^/g)	V_T_ (cm^3^/g)
minimum	100	2.5	14.0	0.3	5.9	20.19	0.42	0.01	0.04	0.010	0.001
maximum	900	20.0	99.9	76.6	12.3	95.30	7.25	46.80	10.21	490.80	1.323
median	460	7.0	37.0	11.1	8.7	69.30	2.97	13.34	1.47	15.00	0.035
mean	470	7.9	44.4	18.2	8.9	65.48	3.11	17.27	1.92	62.60	0.231
mode	700	7.8	46.0	1.1	8.7	89.00	1.20	18.30	0.08	0.001	0.020

η: Yield (%), A: ash content (%), pH: potential of hydrogen (-), C: carbon (%), H: hydrogen (%), O: oxygen (%), N: nitrogen (%), S_BET_: specific surface area (m^2^/g), V_T_: total pore volume (cm^3^/g).

**Table 5:** Biochar characteristics after pyrolysis.

Characteristic	Description	References
Structure	The pyrolysis and carbonation process provide the biomass with a certain degree of aromaticity and a porous structure that is linked to the cellulose and lignin content contained in the material that is pyrolysed. As the temperature increases, the degree of aromaticity and the size of the micropores increase. At temperatures above 700 °C, the carbonaceous structure of the biochar can become unstable.	[35,114,115]
Elemental composition	The main elements in biochar are C, H, O, N, S, P, K, Mg, Na, and Si. The most abundant element is C followed by H and O, while the mineral elements are mainly found in the ashes of the pyrolysis process. The carbon structure is mainly aromatic and can contain acid compounds, alcohols, phenols, esters, and humic and fulvic acids. Nitrogen is mainly present on the surface but is hardly available, while the availability of phosphorus, which is already very low in percentage, decreases as the pyrolysis temperature increases, and this can influence the pH value. The amount of K, Ca, Mg, and Na differs according to the feedstock.	[116,117,118]
Potential of hydrogen (pH)	Biochar contains ash, carbonates, and phosphates due to the pyrolysis process that make biochar alkaline. Generally, the pH increases with pyrolysis temperature due to the volatilisation of organic acids and the decomposition of acidic functional groups.	[35,116,119]
Specific surface area (S_BET_)	The specific surface area of biochar shows a large range (1.5–500 m^2^/g) and increases with temperature due to the decomposition of volatile substances. A reduction in the size of the pores and an increase in their number with a consequent increase in the specific surface is observed. Above a critical temperature, the structure of the micropores collapses, and there is a decrease in the specific surface.	[120,121,122]
Surface functional group	Biochar is characterised by the presence of many functional groups such as carboxyl, carbonyl, and hydroxyl which may contain oxygen or have an alkaline nature. Their presence and their number are linked to the pyrolysis temperature. When the temperature increases overall, their number decreases even if, in percentage, it increases that of the alkaline groups.	[123,124]
Cation exchange capacity (CEC)	The CEC increases during the pyrolysis process due to incomplete decomposition of cellulose. Older biochar can develop additional functional groups that contain oxygen that can increase the O/C ratio and therefore the CEC. Even an increment in K, Ca, or Mg with temperature can cause an increase in the CEC even if sometimes it was observed that an increase in temperature can destroy the functional groups and may lead to a decrease in the CEC.	[125,126]
Stability	Biochar is characterised by low solubility, a high boiling point, and strong resistance to decomposition, so it is very stable in soil.	[127,128]
Water-holding capacity	A decrease in water-holding capacity of biochar with temperature was observed due to the decrease in functional groups and to the increase in aromatisation and the hydrophobicity of biochar.	[129]

**Table 6:** The composition (on a dry basis) of biochars produced from various feedstocks before and after slow pyrolysis, as adapted from Wijitkosum and Jiwnok [87].

Properties	Corncob	Cassava Rhizome	Cassava Stem
C (wt%)	41.66→81.35	37.60→64.25	41.55→62.95
H (wt%)	6.84→2.42	6.15→2.73	6.04→2.24
N (wt%)	0.74→1.22	0.88→1.65	1.27→1.37
O (wt%)	50.76→15.23	55.37→31.80	51.14→33.44
H/C (wt%)	1.97→0.36	1.96→0.43	1.74→0.42
O/C (wt%)	0.91→0.14	1.10→0.37	0.92→0.39
C/N (wt%)	65.63→80.60	50.16→45.72	39.30→54.08
S_BET_ (m^2^/g)	2.54→56.35	2.78→18.38	2.51→200.46
V_T_ (cm^3^/g)	0.0034→0.0405	0.0043→0.0284	0.0058→0.1219
d (Å)	31.05→28.72	69.57→61.69	83.34→24.35

**Table 7:** Main methodologies for the modification of biochar, adapted from Rajapaksha et al. [76] and Tan et al. [56].

Modification Method	Mechanism	Reference
Chemical Oxidation	Introduction of an acidic functional group on the biochar surface.	[134]
H_2_O_2_ Modification	Increased carboxyl groups.	[94]
KOH Modification	Increase in surface area and change in porous texture.	[135]
Physical Activation	Improved the pore structures, higher surface area, larger surface area and pore volume, and increase in sorption capacity.	[136]
Steam Activation	Suppresses CH_4_ and NO_2_ emissions. increases sorption capacity.	[136]
Mineral Addition	Enhanced carbon retention and stability of biochar with mineral treatment due to enhanced formation of aromatic C.	[137]
Magnetic Modification	Significantly improved sorption capacity.	[138]

**Table 8:** Different modifications, residence time (t), and temperature (T) to produce activated biochar.

Biochar	Modification	t (min)	T (°C)	References
Peanut hull hydrochar	10% H_2_O_2_	120	22 ± 0.5	[94]
Iron oxide nanoneedle-array-decorated	Hydrothermal reaction (FeCl_3_ and Na_2_SO_4_) Ultrasonication Autoclave	30 360	n.d. 120	[96]
Beech charcoal	Sessile colonisation (strain 15A)	5760	50	[102]
Water-hyacinth-based BC	KMnO_4_ + 30%H_2_O_2_	30, 720	25, 105	[103]
Hickory wood	5 M NaOH, 0.1 M HCl	240, 120	70, 110–600	[104]
Brewers draff BC	2 M KOH, 0.1 M NaOH/HCl	60	n.d., 105	[139]
Peanut biochar	1 mol/L HCl	24 × 60	60	[95]
	Autoclave	20	121	
SRN-IB 40	Immobilisation and SNR particles	24 × 60	60–70	
Cotton stalk biochar	1 M HNO_3_ and 1 M NaOH	240	RT	[140]

**Table 9:** Main characteristics of heavy metals and concentration limit (mg/L) for drinking water as reported by WHO [1].

Heavy Metal (HM)	Characteristics	References	Limit Concentration (mg/L)
Arsenic (As)	Arsenic compounds in groundwater can be inorganic and organic. Among inorganic compounds, H_3_AsO_3_ for As(III) and H_2_AsO_4_ for As(V) are more common and are toxic species.	[13,18,148,154,155,156,157,158,159,160,161,162,163,164,165]	0.010
Cadmium (Cd)	Cd is often found in combination with zinc (Zn) in carbonate and sulphide ores. Cd(II) is the main oxidation state in groundwater, and its solubility depends on the pH. It remains in solution when the pH is acid and under oxygenated conditions, while it is retained as a mineral in basic conditions.	[7,166,167,168,169,170,171,172,173]	0.003
Copper (Cu)	Among all Cu species, Cu(II) is the most toxic specie because of its stability and solubility in the water. In aerobic alkaline environments, CuCO_3_ is the main soluble species, while in anaerobic environments, it is CuS (s).	[7,11,142,167,174,175]	2.000
Chromium (Cr)	Cr exists in nature mainly in two oxidation states Cr(III) and Cr(VI); however, Cr(III) is more prevalent under reducing conditions, while Cr(VI) is while under oxidising conditions. Cr is rarely detected in non-contaminated groundwater.	[167,176,177,178,179,180,181]	0.050
Manganese (Mn)	This metal exists in water in four oxidation states (Mn(II), Mn(IV), Mn(VI), and Mn(VII)) depending on the physicochemical properties of the water.	[167,182,183,184,185,186,187]	0.400
Mercury (Hg)	Mercury, when in free form, is insoluble in water. Among the forms, inorganic Hg(II) ions are particularly stable when combined with sulphur ions. It can also transform into organomercuric forms such as methylmercury (MeHg).	[188,189]	0.006
Nickel (Ni)	This metal presents two main oxidation states, Ni(II) and Ni(III); however, Ni(II) is the most common in biological systems.	[167,190,191,192,193]	0.070
Lead (Pb)	It is a heavy metal whose extensive use has caused environmental contamination and health problems in many parts of the world.	[194,195,196,197,198]	0.010
Zinc (Zn)	Zn is present in water as inorganic and organic complexes, and colloids.	[167,199,200,201]	not regulated

**Table 10:** Heavy metals (HMs) and interaction with biochar.

HM	Effects	References
Cr	Biochar interacts with chromium, favouring the transformation from Cr(VI) to Cr(III). This process can be facilitated by biochar which has a suitable number of functional groups that can be obtained with suitable feedstock and slow pyrolysis.	[108]
Cd	Experimental studies show that Cd is then subjected to an absorption process by cation exchange which increases with increasing pH.	[257]
Pb	The main mechanism for Pb sorption is precipitation. If the temperature increase, the sorption of Pb increases due to the mineral composition of biochar.	[258]
Hg	The main mechanisms for Hg sorption are action exchange, complexation, and precipitation. The sorption mechanism is strongly dependent on biochar characteristics (feedstock, pyrolysis temperature, and pH).	[259]
As	The main mechanisms for As sorption are complexation and chemical reduction.	[260]

**Table 11:** Heavy metals and different kinds of biochar, adapted from Ahmad et al. [111].

HM	Biochar	T (°C)	Effect
Cr	Oak wood	400–450	Sorption
	Oak bark		
Cr	Sugar beat tailing	300	Electrostatic attraction; reduction of Cr(VI) to Cr(III); complexation
Cu	Crop straw	400	Adsorption due to surface complexation
Cu	Pecan shell	800	Sorption on humic acid at pH equal to 6; precipitation of azurite or tenorite at pH 7, 8 and 9
Cu and Zn	Hardwood	450	Endothermic adsorption
Pb	Dairy manure	200	Precipitation with phosphate
	Sewage sludge	550	Adsorption due to cation release and functional groups complexation on surface
Hg	Soybean stalk	300–700	Precipitation, complexation, and reduction

**Table 12:** Effects of feedstock proprieties, initial concentration (C_0_), and bed volume (BV) on heavy metal (HM) removal from biochar.

Biochar	HM	C_0_	BV	R (%)	q (mg/g)	Ref.
Straw	As	10 µg/L	n.d.	50.0	n.d.	[93]
		50 µg/L		80–90		
		100 µg/L		50–90		
		10 µg/L		100		
		50 µg/L		90		
Peanut hull hydrochar	Pb	50 mg/L	3–400	0–100	1.04	[94]
H_2_O_2_ peanut hull hydrochar	Pb	50 mg/L	150–400	100–50	22.82	
	Ni	14.68 mg/L	40–400	n.d.	0.07	
	Cd	28.11 mg/L		n.d.	0.21	
	Cu	49.25 mg/L		10–100	1.22	
	Pb	51.80 mg/L		20–100	16.45	
Fe-NN/BFs	As(V)	275 µg/L	20	n.d.	93.94	[96]
	As(III)				70.22	
Dairy manure	Pb	50.0 mg/L	n.d.	97.4	10.1	[98]
	Cu	50.0 mg/L		53.4	4.75	
	Zn	1.50 mg/L		54.5	109 × 10^3^	
Beech charcoal	Cd	25 mg/L	n.d.	n.d.	0.79	[102]
Beech bio-charcoal					3.19	
AMBIOTON^®^	Pb	100 mg/L	n.d.	n.d.	110.73	[99]
RE-CHAR^®^					163.89	[101]
Hardwood biochar	As	96	n.d.	>99	n.d.	[59]
	Cd	119				
	Zn	249 mg/L				
BC@MnO_2_-X	Pb	75 mg/L	320	>98	2.1 × 10^6^	[103]
	Cd	60 mg/L	233		1.1 × 10^6^	
	Cu	40 mg/L	267		6.7 × 10^5^	
	Zn	40 mg/L	213		5.2 × 10^5^	
HB	Pb Cu Cd Zn Ni	100 mg/L	n.d.	n.d.	3.32 2.64 0.71 0.20 0.24	[104]
HBM					19.1 17.9 1.83 0.98 0.89	
Acacia confusa and Celtic sinensis biochar	Cu	2–500 mg/L	40–220	n.d.	7.51	[100]
	Zn		40–70		2.44	
	Pb		220–450		10.2	
Brewers draff	Cu	1.15 mg/L	n.d.	60.8–55.6	8.77	[105]
Brewers draff + KOH				60.0–51.3	10.30	
CBB	Cd	50 mg/L	n.d.	36	192–123	[106]
	Cu			25	210–156	
	Zn			48	178–92	
Sewage sludge biochar	Cr	100 mg/L	n.d.	99.77	5.724	[107]
	Mn	100 mg/L		100.00	5.681	
	Cu	50 mg/L		99.98	5.342	
	Zn	250 mg/L		99.99	5.905	
Peanut shell biochar	Cr	70 mg/L	n.d.	100	25.1	[95]

HM: Heavy metals, C_0_: initial concentration (mg/L or µg/L), BV: bed volume, R: removal efficiency (%), q_m_: maximum adsorption capacity (mg/g or µg/g).

**Table 13:** Effects of biochar and type of activation on physical properties and adsorption capacities (q_m_).

Biochar	Pyrolysis Process	Activation Type	Physical Characterisation	HM	q_m_ (mg/g)	Reference
Raw cypress sawdust (RCS)	n.d.	n.d.	S_BET_: 10.5 m^2^/g	Pb	7.4–7.9	[250]
		Pretreated with magnesium	S_BET_: 35.0 m^2^/g		77.1–97.2	
Bovine bones (BB)	n.d.	n.d.	S_BET_: 113.3 m^2^/g V_mi_: 0.001 cm^3^/g	Cd	94.43–5.81 × 10^2^	[263]
				Ni	1.77–1.18 × 10^2^	
				Zn	15.70–2.86 × 10^2^	
				Cu	4.23 × 10^2^–1.06 × 10^3^	
Cotton fibre (BF)	T: 800 °C	n.d.	S_BET_: 2.45 m^2^/g	As(III)	70.22	[96]
	t: 2 h	With iron oxide nanoneedles	S_BET_: 8.68 m^2^/g	As(V)	93.94	
Rice husk (RH)	T: 700 °C	n.d.	S_BET_: 37.04 m^2^/g V_T_: 0.03 cm^3^/g	As(III)	0.04	[251]
	t: 8 h	Activated by MnO_2_	S_BET_: 81.73 m^2^/g V_T_: 0.13 cm^3^/g	As(V)	3.49	

HM: Heavy metals, t: time (h), T: temperature (°C), S_BET_: specific surface area (m^2^/g), V_mi_: micropore volumes (cm^3^/g), V_T_: total pore volume (cm^3^/g), q_m_: maximum adsorption capacity (mg/g or µg/g).

**Table 14:** Effects of chemical properties of biochar on maximum adsorption capacity and removal efficiency.

Feedstock	Pyrolysis Process	Activation	Chemical Properties	HM	q_m_ (mg/g)	R (%)	Ref.
Peanut shell (PHHC)	n.d.	PHHC activated with hydrogen peroxide H_2_O_2_ (mPHHC)	PHHC C: 56.3% H: 5.6% O: 36.6%	Ni Cd Cu Pb	PHHC Pb Monometallic 1.04	n.d.	[94]
			mPHHC C: 48.3% H: 5.8% O: 43.8%		mPHHC Pb Monometallic 22.82 multimetallic 16.45		
Citrus maxima peel (CM) Passion fruit shell (PF) Sugar cane bagasse (SB)	T: 50 °C t: 18 h	n.d.	CEC (m_eq_/100 g) 47.3 (CM) 26.9 (PF) 11.8 (SB)	Cu Cd Ni Pb	11.63–46.69 6.64–26.65 3.47–13.92 12.23–49.11	n.d.	[249]
Exhausted mushroom compost (SMCB)	T: 500 °C t: 3 h	n.d.	A: 50.6% VM: 13.8% C: 35.94% H: 1.21% N: 2.18% O: 38.83% CEC: 57.6 mmol/kg	Zn Cu Pb	0.60 1.11 12.7	n.d.	[146]
Sewage sludge from a municipal water treatment plant (SDBC)	T: 600 °C t: 2 h	Activated by impregnation in liquid phase of nano-zero-valent iron (nZVI–BC)	n.d.	Cr	23.5–39.8	n.d.	[265]
Crop residues of Chenopodium quinoa (QBC)	T: 400 °C t: 1 h	Activated with magnetite nanoparticles (QBC/MNP) and with strong acid HNO_3_ (QBC/Acid)	Crystal size (QBC/MNP, QBC/Acid, QBC) 12.4 nm, 42.6 nm, and 63.5 nm.	Cr	n.d.	QBC 48.85–75.28 QBC/MNP 73.35–93.62 QBC/Acid 55.85–79.8	[266]

HM: Heavy metal, t: time (h), T: temperature (°C), q_m_: maximum adsorption capacity (mg/g or µg/g), R: removal efficiency (%), A: ash content (%), VM: volatile matter (%), C: carbon (%), H: hydrogen (%), O: oxygen (%), N: nitrogen (%), CEC: cation exchange capacity (mmol/kg).

**Table 15:** Different column-scale experimental setup and operation parameters.

Material	h (cm)	ϕ (cm)	Q (mL/min)	z	Data Collection Time	References
Plexiglas	14.5	4.5	1.27	3.0 cm	11 h	[93]
Acrylic	5.1	1.56	1.00	2.1 cm	n.d.	[94]
n.d.	10	1.2	2.26	2.0 g	n.d.	[96]
Polypropylene	20	10	1.75–2.25	5% (*w*/*w*)	1.0 L	[98]
Glass	18	1.0	1.00	0.5 g	1 h	[102]
Glass	18	1.0	0.40	6.0 cm 0.1 g	1 h	[99,101]
Glass	20	5.0	0.10	15 cm	10 mL	[59]
Polyethylene	13	1.5	1.00	0.3 cm 4.5 g	120 mL	[103]
Acrylic	n.d.	0.5	2.00	1.55 cm	2 min	[104]
Polyethylene	6.8	1.5	1.30	10 wt%	n.d.	[100]
Glass	13	2.0	5.00	3.0 g	n.d.	[105]
Plexiglas	30	5.0	0.40	5% (*w*/*w*)	100 mL	[107]
PVC cylinder	n.d.	2.5	0.020	9.1 g	n.d.	[106]
Plexiglas	40	5.0	n.d.	n.d.	30 min	[95]

**Table 16:** Description of the system in the column and of the operating conditions.

Biochar	HM	Column Characteristics	Layers	Operating Conditions	q_m_ (mg/g)	R (%)	Ref.
Biochar from agricultural organic waste (CM-PF-SB)	Pb Cd Cu Ni	ϕ: 1 cm h: 10 cm	z: 3 cm	T: 25 °C Q: 2.0, 3.0 and 4.0 mL/min	173.134, 101 144.108, 72.3 98,70, 45.6 70.56, 35.0	n.d.	[249]
Biochar from cypress sawdust (RCS)	Pb	Plexiglas ϕ: 2.9 cm h: 20 cm σ: 6.6 cm^2^	Uniform layering with small increments of RCS or Mg-B biochar. Glass particles at the end of the column.	Q (mL/min) 79 59 45	7.5 7.9 8.8	n.d.	[250]
RCS magnetised with Mg (Mg-B)					85.5 97.1 120.7		
Biochar from agricultural waste	As	Plexiglas ϕ: 4.5 cm h: 14.5 cm	Two layers of fine saturated sand (4 cm at the top and 4 cm at the bottom) and a layer of gravel (2 cm at the bottom and 1.5 cm at the top). In the centre z: 3.0 cm	C_0_: 100 µg/L Steady-state experiments. Water drawn from cassettes through capillaries (internal diameter: 2.5 mm).	n.d.	ASW 100%	[93]
						GW 95%	
Biochar from cotton fibres (BF)	As(III) As(V)	ϕ: 12 mm h: 100 mm	10 mm of quartz wool at the end. w: 9.04 mL	RT. Upward flow in the column. A peristaltic pump.	n.d.	n.d.	[96]
BF magnetised with Fe (Fe-NN/BFs)					70.22 As(III) 93.94 As(V)		
BRH-MnO_2_		Glass ϕ: 2.7 cm h: 30 cm	fixed-bed column 3 cm of quartz sand at end of column.	Hybrid system with electro absorption. Q: 5, 10 and 15 (mL/min)	2.88 2.75 2.62	n.d.	[251]

HM: Heavy metal, h: heigh of the column (cm), ϕ: diameter of column (cm), z: depth of the adsorbent bed (cm, g or wt%), Q: flow rate (mL/min), q_m_: maximum adsorption capacity (mg/g or µg/g), R: removal efficiency (%).

**Table 17:** Effects of biochar dosage on adsorption capacity and removal efficiency.

Biochar	Dosage	Heavy Metal	q_m_ (mg/g)	R (%)	References
RCS	m: 5.0, 10.0 and 13.7 g	Pb	6.9 7.9 8.4	n.d.	[250]
RCS-Mg-B			94.5 97.1 97.0		
BCM	m/m_s_: 50 g/kg	As(V)	n.d.	33.7	[269]
		Cr(VI)		77.3	
WSB	m/v: 0.5–8.0 g/L	Cd	40.0	35–62	[270]
AWSB			60.0	61–86	
BCS	4% of m_s_	Cd	n.d.	16.2–28.7	[271]
		Ni		22.2–57.2	

HM: Heavy metal, m_s_: mass of the soil, m/v: dosage of biochar or mass per unit of volume (g/L), m/m_s_: dosage of biochar or mass per unit of weight (g/kg), q_m_: maximum adsorption capacity (mg/g or µg/g), R: removal efficiency (%).

**Table 18:** Waters, aqueous solutions, and real samples of contaminated groundwater used for heavy metal removal studies using biochar.

Water	Heavy Metals	C_0_	Ref.
Aqueous solution	As	10, 50, 100 µg/L	[93]
Real contaminated groundwater		5, 10, 50 µg/L	
Aqueous solution	Pb	50 mg/L	[94]
Mixture solution	Ni	14.68 mg/L	
	Cd	28.11 mg/L	
	Cu	49.25 mg/L	
	Pb	51.80 mg/L	
Spiked natural groundwater	As(III)	275 µg/L	[96]
	As(V)		
Spiked natural groundwater	Pb	50 mg/L	[98]
	Zn	50 mg/L	
	Cd	1.5 mg/L	
Contaminated solution	Pb	100 mg/L	[99,101]
Leaching from a sediment-derived soil	As	96–280 mg/kg	[272]
	Cd	142– 862 mg/kg	
	Zn	789–930 mg/kg	
Contaminated water	Cd	25 mg/L	[102]
Mixed-metal solution	Pb	100 mg/L	[104]
	Cd		
	Cu		
	Zn		
	Ni		
Stock solutions	Cu	2–500 mg/L	[100]
	Zn		
	Pb		
Soil solution	Cu	1.15 mg/L	[105]
Nutrient medium	Cr	50 mg/L	[95]

**Table 19:** Effects of heavy metal and different initial concentrations on adsorption capacity.

Biochar	HM	Chemical Compound	C_0_	R (%)	q_m_ (mg/g)	Ref.
RCS	Pb	Pb(NO_3_)_2_ in DI	25 mg/L 50 mg/L 100 mg/L	7.4 7.8 7.9	n.d.	[250]
RCS-Mg-B				77.1 81.9 97.2	n.d.	
CMBC			150 mg/L	n.d.	5.80	[274]
BAW	As(III) As(V)	Na_2_HAsO_4_ + 7H_2_O on DI	10 mg/L	50	n.d.	[93]
			50 mg/L	90		
			100 mg/L	90		
		CGW	5 mg/L	100	n.d.	
			10 mg/L	100		
			50 mg/L	90		
BRH-MnO_2_		NaAsO_2_ As_2_O_5_	1.0 mg/L	n.d.	2.88	[251]
			2.0 mg/L		3.13	
			5.0 mg/L		3.49	
Biochar from wheat straw (WSB)	Cd	Cd(NO_3_)_2_ in DI	25 mg/L 50 mg/L 100 mg/L	89.20–93.35 (t: 90 min) 71.60–77.50 (t: 240 min)	n.d.	[270]
Biochar from wheat straw and acidified with H_3_PO_4_ (AWSB)				92.30–95.60 (t: 90 min) 78.40–86.28 (t: 240 min)		
Biochar modified with MgCl_2_ derived from shrimp shell waste (MgC600)		n.d.	50 mg/L	n.d.	8.14	[275]
			100 mg/L		7.73	
			150 mg/L		8.44	
Quinoa biochar (QBC)	Cr	n.d.	50 mg/L 100 mg/L	60.62 53.60	n.d.	[266]
Magnetised quinoa biochar (QBC/MNPs)				5.48 77.50		
Acidified quinoa biochar (QBC/Acid)				75.24 69.84		
Biochar from barley grass (BC) and magnetised with Fe (Fe-BC)		Contaminated GW prepared by leaching DI from contaminated soil	Soil 628 mg/kg Cr(III) 6622 mg/kg Cr(VI) GW 126 mg/L Cr(III) 701 mg/L Cr(VI)	Fe-BC: 78 Cr(III) Fe-BC: 22 Cr(VI) Fe-BC: 70.6 Cr(tot)	n.d.	[276]

HM: heavy metal, t: time (h), q_m_: maximum adsorption capacity (mg/g or µg/g), C_0_: initial concentration (mg/L or µg/L), R: removal efficiency (%).

**Table 20:** Effects of heavy metals and pH variation on adsorption capacity.

Feedstock	pH	HM	R (%)	q_m_ (mg/g)	Ref.
agricultural organic waste (CM, PF, and SB)	4.0 5.0 6.0	Pb	n.d.	126 163 169	[249]
		Cd		116 125 132	
		Cu		72.0 82.2 84.0	
		Ni		52.7 59.3 60.7	
spent mushroom compost (SMCB)	8.8	Zn Cu Pb	n.d.	0.60 1.11 12.7	[146]
raw cypress sawdust (RCS) and magnetised with Mg (Mg-B)	RCS: 5.7 Mg-B: 9.7	Pb	n.d.	6.9–8.4 77.1–97.2	[250]
PHHC	6.2		n.d.	1.04	[94]
mPHHC	4.4		n.d.	22.82	
		Cd Cu Ni Pb	n.d.	0.21 1.22 0.07 16.45	
wheat straw (WSB) and acidified with H_3_PO_4_ (AWSB)	2–6	Cd	35–82 45–98	16–41 23–48	[270]

HM: heavy metal, q_m_: maximum adsorption capacity (mg/g or µg/g), R: removal efficiency (%).

**Table 21:** Parameters for the description of adsorption obtained by means of pseudo-first- (PFO) and -second-order kinetic models (PSO).

Biochar	HM	Kinetic Models	Parameters	Ref.
PHHC	Ni Cd Cu Pb	PFO PSO Elovich	R^2^: 0.86 R^2^: 0.89 R^2^: 0.89	[94]
mPHHC			R^2^: 0.90 R^2^: 0.96 R^2^: 0.99	
Biochar from date seeds	Pb	PFO	k_PFO_: 0.011 1/min q_e_: 9.117 mg/g R^2^: 0.86	[280]
		PSO	k_PSO_: 3.007 × 10^−3^ g/(mg min) q_e_: 9.117 mg/g R^2^: 0.94	
Biochar from cotton fibres (BF) Biochar from cotton fibres and magnetised with Fe (Fe-NN/BFs)	As(III) As(V)	PFO PSO	q_e_: 70.22 mg/g As(III) q_e_: 93.94 mg/g As(V)	[96]
Biochar from rice husk (BRH)		PFO PSO	R^2^ > 0.80 q_e_: 0.18 mg/g As(III) q_e_: 0.44 mg/g As(V)	[251]
Biochar activated by MnO_2_ (BRH-MnO_2_)			R^2^ > 0.992 q_e_: 1.36 mg/g As(III) q_e_: 2.12 mg/g As(V)	
Quinoa biochar (QBC), magnetised with Mn (QBC/MNP) and acidified HNO_3_ (QBC/Acid)	Cr(VI)	PFO	q_e_: 6.67 mg/g	[266]
			q_e_: 5.29 mg/g	
			q_e_: 5.38 mg/g	
		PSO	q_e_: 16.37 mg/g R^2^: 0.99	
			q_e_: 12.77 mg/g R^2^: 0.99	
			q_e_: 15.22 mg/g R^2^: 0.99	

**Table 22:** Models and related parameters for the description of breakthrough curve data.

Biochar	HM	Models	Operating Conditions	Parameters			Ref.
Biochar from rice husks (BC) and activated by MnO_2_ (BC active)	As(III) As(V)	Thomas	C_0_ (mg/L) 1 2 5 Q (mL/min) 5 10 15	k_th_ (L/mg min) 0.0030 0.0025 0.0016 0.0025 0.0044 0.0057	q_m_ (mg/g) 2.88 3.13 3.49 2.88 2.75 2.62	R^2^ 0.938 0.932 0.943 0.938 0.966 0.900	[251]
Biochar from sewage sludge (BC) and activated with iron (nZVI-BC)	Cr(VI)	Thomas	C_0_ (mg/mL) 0.1–0.05 0.05–0.2 Q (mL/min) 0.5–1.0 1.0	k_th_ (L/mg h) 0.036–0.1 0.1–0.03	q_m_ (mg/g) 39.85–26.65 26.65–27.67	R^2^ 0.993–0.995 0.995–0.994	[265]
		Yoon–Nelson		k_YN_ (1/h) 0.2–0.31 0.31–0.37	τ (h) 15.94–10.6 10.6–2.7	R^2^ 0.993–0.995 0.995–0.994	
Biochar from peanut shells (PHHC) and activated with H_2_O_2_ (mPHHC)	Pb	ADE	PHHC mPHHC	k_ADE_ (1/min) 1.29 3.68	q_m_ (mg/g) 1.04 22.82	R^2^ 0.78 0.98	[94]
	Pb Cd Cu Ni		mPHHC Cd Cu Ni Pb	k_ADE_ (1/min) 4.26 106 8.17 0.75	q_m_ (mg/g) 0.21 1.22 0.07 16.45	R^2^ 0.99 0.95 0.99 0.95	
Biochar from cypress sawdust (RCS) and magnetised with Mg (Mg-B)	Pb	CXTFIT	C_0_: 50–100 mg/L Q: 45–79 mL/min h: 20 cm ϕ: 2.9 cm	u (cm/min) 11.5 12.9	x (cm) 0.34 0.37		[250]
		Hydrus 1D		R^2^ (RCS) 0.996 0.982 0.994 0.991	R^2^ (Mg-B) 0.981 0.998 0.986 0.973		
Biochar from date seeds		Bohart–Adams	h: 50 cm ϕ: 2.5 cm C_0_: 103.6 mg/L Q: 1.0 mL/min	n.d.	R^2^ 0.76		[280]
		Thomas		n.d.	R^2^ 0.77		
		Modified dose response		q_max_ (mg/g) 4.538 × 10^2^	R^2^ 0.95		
Spent mushroom compost (SMCB)	Zn Cu Pb	Thomas	C_0_ (mg/L) 196.17 Zn 590.90 Cu 621.60 Pb Q: 1.6 mL/min	k_th_ (mL/min mg) 2.55 3.19 0.26	q_m_ (mg/g) 0.6 × 10^4^ 1.11 × 10^4^ 12.7 × 10^4^	R^2^ 0.983 0.991 0.985	[146]
		Clark		B (min) 6.379 6709 2331	r (mg/L min) 0.028 0.056 0.014	R^2^ 0.989 0.981 0.976	

HM: heavy metal, q: maximum adsorption capacity (mg/g or µg/g), C_0_: initial concentration (mg/L or µg/L), h: heigh of the column (cm), ϕ: diameter of column (cm), Q: flow rate (mL/min), q_m_: maximum adsorption capacity (mg/g or µg/g), R^2^: coefficient of determination (-), u: linear velocity (cm/min), k_th_: constant of the Thomas model [mL/(min mg)], k_ADE_: constant of the advection–dispersion equation (1/min).

## Data Availability

Data are contained within this review.

## Abbreviations

a	constant of modified dose-response model
A	ash content (%)
AC	activate carbon
ADE	advection–diffusion equation
ATSRD	Agency for Toxic Substances and Disease Registry
AWSB	biochar from wheat straw and acidified with H_3_PO_4_
ASW	aqueous solution
B	Clark model constant (min)
BA	Bohart–Adams model
BAW	biochar from agricultural waste
BRH	biochar from rice husks (BC)
BC	biochar
BC@MnO_2_-X	biochar loaded with manganese dioxide
BRH-MnO_2_	biochar from rice husks and activated by MnO_2_
BCact	activated biochar
BCM	biochar from chicken manure
BCS	biochar from corn straw
BB	bovine bones
BF	biochar from cotton fibres
BRH	biochar from rice husks
c	carbon (%)
C	concentration at time t or output concentration (mg/L or µg/L)
C_0_	initial concentration (mg/L or µg/L)
CBB	chicken bone biochar
CDI	capacitive deionisation
CEC	cation exchange capacity (mmol/kg)
CF	cotton fibres
CGW	contaminated groundwater
CM	biochar from agricultural organic waste
CMBC	Chitosan-modified biochar
CNEM	chemical non-equilibrium model
CSB	cotton stalk biochar
d	average pore diameter (Å)
D	average particle size (mm or µm)
DI	deionised water
DOM	dissolved organic matter
EDS	X-ray spectroscopy
EU	European Union
EXAFS	X-ray absorption spectroscopy
FAO	Food and Agriculture Organization of the United Nations
FC	fixed carbon (%)
FeNN/BF	iron oxide nanoneedle-array-decorated biochar Fibres
Fe-NN/BF	biochar from cotton fibres magnetised with Fe
FTIR	Fourier-transform infrared spectroscopy
h	height of the column (m or cm)
H	hydrogen (%)
HB	hemp-derived biochar
HM	heavy metals
IARC	International Agency of Research on Cancer
IUPAC	International Union of Pure and Applied Chemistry
k_ADE_	constant of advection-dispersion equation (1/min)
k_BA_	constant of the Bohart–Adams model [L/(mg min)]
k_PFO_	constant of the pseudo-first-order model (1/min)
k_PSO_	constant of the pseudo-second-order model ([g/(mg min)]
k_Th_	constant of the Thomas model [mL/(min mg)]
k_YN_	constant of the Yoon–Nelson model (1/min)
m	mass of the adsorbent (g)
M	moisture content (%)
m_s_	mass of the soil (Kg)
m/m_s_	dosage of biochar or mass per unit of weight (g/kg)
m/v	dosage of biochar or mass per unit of volume (g/L)
Mg-B	biochar from raw cypress sawdust and magnetised with Mg
mPHHC	biochar hydrothermally produced from peanut shells and activated with hydrogen peroxide H_2_O_2_
mPHHC	biochar from peanut shells and activated with H_2_O_2_
MDR	modified dose-response model
MTZ	mass transfer zone
*n*	Freundlich constant (-)
N	nitrogen (%)
NMR	nuclear magnetic resonance
n.d.	not detected
nZVI-BC	activated biochar with impregnation of nano-zero-valent iron
O	oxygen (%)
*p*	porosity (-)
PF	biochar from agricultural organic waste
PFO	pseudo-first-order
pH	potential of hydrogen (-)
pH_PZC_	point of zero charge (-)
PHA	polycyclic aromatic hydrocarbons
PHHC	biochar from peanut shells
PRB	permeable reactive barrier
PSO	pseudo-second-order
q_e_	equilibrium adsorption capacity (mg/g or µg/g)
q_m_	maximum adsorption capacity (mg/g or µg/g)
q_t_	adsorption capacity at time t (mg/g or µg/g)
Q	flow rate (mL/min)
QBC	quinoa biochar
QBC/MNP	quinoa biochar/magnetic nanoparticle
r	Clark model constant (mg/L min)
R	removal efficiency (%)
R^2^	coefficient of determination (-)
RB	raw biochar
RCS	biochar from cypress sawdust
RCS-Mg-B	biochar from cypress sawdust and Mg magnetised particles
RDA	recommended dietary allowance (mg/d)
RH	rice husk
RT	room temperature
Sb	biochar from agricultural organic waste
S_BET_	specific surface area (m^2^/g)
SDBC	biochar produced by sewage sludge from a municipal water treatment plant
SEM	scanning electron microscopy
SMBC	biochar from spent mushroom compost
SMCB	biochar from spent mushroom compost
SOM	soil organic matter
SS	sewage sludge
STEM	scanning transmission electron microscopy
t	time (d, h, min, or s)
t_b_	breakpoint time (h or min)
t_e_	exhaustion time (h or min)
T	temperature (°C)
TEM	transmission electron microscopy
u	linear velocity of influent solution (cm/min)
u_i_	components of the velocity vector (cm/min)
V_mi_	micropore volumes (cm3/g)
V_T_	total pore volume (cm^3^/g)
VM	volatile matter (%)
w	volume of the adsorbent bed (cm^3^ or mL)
x	direction of motion or axial dispersion (cm)
YN	Yoon–Nelson model
WHO	World Health Organization
WSB	biochar from wheat straw
XPS	X-ray photoelectron spectroscopy
XRD	Xray diffraction
z	depth of the adsorbent bed (cm, g or wt%)
z_s_	depth of the layer sand (cm)
η	yield (%)
λ	longitudinal dispersion coefficient (-)
ρ	average mass density (g/cm^3^)
σ	column cross section (cm^2^ or mm^2^)
τ	time required for the passage 50% of the adsorbate (min)
ϕ	diameter of the column (cm or mm)
